# Prevalence of *PALB2* mutations in Australian familial breast cancer cases and controls

**DOI:** 10.1186/s13058-015-0627-7

**Published:** 2015-08-19

**Authors:** Ella R. Thompson, Kylie L. Gorringe, Simone M. Rowley, Michelle W. Wong-Brown, Simone McInerny, Na Li, Alison H. Trainer, Lisa Devereux, Maria A. Doyle, Jason Li, Richard Lupat, Martin B. Delatycki, LifePool Investigators, Gillian Mitchell, Paul A. James, Rodney J. Scott, Ian G. Campbell

**Affiliations:** Cancer Genetics Laboratory, Peter MacCallum Cancer Centre, Locked Bag 1, A’Beckett St, East Melbourne, VIC 8006 Australia; The Sir Peter MacCallum Department of Oncology, St Andrews Place, East Melbourne, VIC 3002 Australia; Department of Pathology, University of Melbourne, Corner GrattonStree and Royal Parade, Melbourne, VIC 3010 Australia; Discipline of Medical Genetics and Centre for Information-Based Medicine, The University of Newcastle and Hunter Medical Research Institute, 1 Kookaburra Circuit, Newcastle, NSW 2305 Australia; Familial Cancer Centre, Peter MacCallum Cancer Centre, St Andrews Place, East Melbourne, VIC 3002 Australia; Cancer Biology Research Center, Tongji Hospital, Tongji Medical College, Huazhong University of Science and Technology, 1037 Luoyu Road, Wuhan, Hubei China; LifePool, Peter MacCallum Cancer Centre, St Andrews Place, East Melbourne, VIC 3002 Australia; Bioinformatics Core Facility, Peter MacCallum Cancer Centre, St Andrews Place, East Melbourne, VIC 3002 Australia; Austin Health, 145 Studley Road, Heidelberg, VIC 3084 Australia; Hereditary Cancer Program, BC Cancer Agency, 600 West 10th Avenue, Vancouver, BC V5Z 4E6 Canada; Division of Genetics, Hunter Area Pathology Service, Lookout Road, Newcastle, NSW 2305 Australia

## Abstract

**Introduction:**

*PALB2* is emerging as a high-penetrance breast cancer predisposition gene in the order of *BRCA1* and *BRCA2*. However, large studies that have evaluated the full gene rather than just the most common variants in both cases and controls are required before all truncating variants can be included in familial breast cancer variant testing.

**Methods:**

In this study we analyse almost 2000 breast cancer cases sourced from individuals referred to familial cancer clinics, thus representing typical cases presenting in clinical practice. These cases were compared to a similar number of population-based cancer-free controls.

**Results:**

We identified a significant excess of truncating variants in cases (1.3 %) versus controls (0.2 %), including six novel variants (*p* = 0.0001; odds ratio (OR) 6.58, 95 % confidence interval (CI) 2.3–18.9). Three of the four control individuals carrying truncating variants had at least one relative with breast cancer. There was no excess of missense variants in cases overall, but the common c.1676A > G variant (rs152451) was significantly enriched in cases and may represent a low-penetrance polymorphism (*p* = 0.002; OR 1.24 (95 % CI 1.09–1.47).

**Conclusions:**

Our findings support truncating variants in *PALB2* as high-penetrance breast cancer susceptibility alleles, and suggest that a common missense variant may also lead to a low level of increased breast cancer risk.

## Introduction

Partner and localizer of BRCA2 (PALB2) plays a central role in homologous recombination-mediated repair of double-strand DNA breaks [1] and biallelic mutations are responsible for Fanconi anemia complementation group N [2]. Monoallelic inactivating germline mutations in *PALB2* were subsequently shown to be associated with familial breast cancer [3] and numerous studies supported this association in various populations and established a mutation prevalence of approximately 1 % among familial breast cancer cases (varying from 0.1 % to 2.7 % as reviewed by Southey et al. [4]). Most recently, Antoniou et al*.* used a modified segregation analysis approach to determine that the age-specific risk of breast cancer among female mutation carriers overlaps the risk conferred by *BRCA2* mutations [5] establishing that, despite the rarity of mutations, *PALB2* is the most significant breast cancer predisposition gene after *BRCA1* and *BRCA2*.

In Australia, early studies identified *PALB2* c.3113G > A (p.Trp1038*) as a recurring truncating mutation among familial breast cancer index cases, and established the enrichment of c.3113G > A in cases compared to controls [6]. Further studies have identified a spectrum of truncating variants among breast cancer cases [7–10], the collective frequency of which has not been compared to Australian controls. Indeed few studies of *PALB2* mutations have analysed significant numbers of family cancer clinic-ascertained cases or matched controls. Because early studies focused on screening just for the presumed common pathogenic mutations, in Australia (eviQ Cancer Treatments Online; [11]) it is not recommended to test for *PALB2* truncating mutations aside from the recurring c.3113G > A variant, however, it is likely that all truncating mutations confer an equivalent loss of gene function and consequent breast cancer risk. Other guidelines, such as National Comprehensive Cancer Network [12], have made no specific distinction between different *PALB2* mutations but do raise a general caution around the interpretation of testing for mutations in *PALB2* and other “moderate penetrance” breast cancer predisposition genes, especially as part of panel tests. Identification of genetic risk factors is critical for individual risk assessment and reduction strategies, and in the near future may provide avenues for personalised therapy [4]. Therefore it is important to continue to amass the necessary data to support the implementation of whole gene testing of *PALB2* in breast cancer families. In this study, we performed germline mutation analysis of the entire coding region of *PALB2* in a cohort of 1996 breast cancer index cases referred to familial cancer clinics for genetic testing and tested negative for *BRCA1* and *BRCA2* mutations as well as 1998 Australian cancer-free female controls. This represents the largest single case/control screen of germline *PALB2* mutations to date.

## Methods

### Samples for mutation analysis

Cancer-affected women in the study were referred by their physician to a specialist Familial Cancer Centre (FCC) for genetic testing of *BRCA1* and *BRCA2* between 1997 and 2014, and were identified as being at “high risk” of carrying a predisposing allele. The criteria for high risk included a personal history of breast cancer, two or more first- or second-degree relatives with breast and/or ovarian cancer, and an additional risk factor (additional affected close relatives, diagnosis before 40 years, multiple primary breast or ovarian cancers in one individual, or Ashkenazi Jewish ancestry). From 2003, individuals with a ≥10 % risk of carrying a *BRCA1* or *BRCA2* mutation, as estimated by BRCAPro, including tumour pathology, were also eligible [13].

Our final case cohort (Additional file 1) included 997 breast (95 %) or ovarian (5 %) cancer-affected index cases from the Hunter Area Pathology Service (HAPS), Newcastle, Australia [9]. Family history information was available for a subset of this cohort only. A further 999 breast cancer-affected index cases each with detailed family history available were obtained from the combined Victorian Familial Cancer Centres (FCCs) through the Variants in Practice (ViP) study. For all cases, clinical genetic testing of *BRCA1* and *BRCA2*, including for large rearrangements by multiplex ligation probe-dependent amplification (MLPA), returned negative results.

A cohort of 1998 participants in the LifePool study [14] were utilised as cancer-free population control samples for this analysis. LifePool recruits female participants through the Australian population mammography screening program (BreastScreen) for research studies utilising prospectively collected epidemiological, genetic and mammographic data with ongoing clinical follow-up obtained through the Victorian Cancer Registry. Participants provided breast cancer family history information for close relatives only. The average age of the participants recruited to this study was 58.84 ± 9.9 years (range 19–91).

All cases and controls provided informed consent for genetic analysis of their germline DNA. This study was approved by the Human Research Ethics Committees at each participating ViP centre (see Acknowledgements), the Peter MacCallum Cancer Centre, Hunter New England Health and The University of Newcastle. This study was carried out in accordance with all relevant regulations and guidelines.

### Germline mutation analysis

Germline mutation analysis of the *PALB2* gene was performed as part of a custom sequencing panel. All coding *PALB2* exons were amplified from 225 ng of germline DNA extracted from blood or saliva using the HaloPlex Targeted Enrichment Assay (Agilent Technologies, Santa Clara, CA, USA) according to the manufacturer’s protocol using an Agilent Bravo Automated Liquid Handling System. Paired-end 100 or 150 bp sequence reads were generated from the indexed, pooled libraries on a HiSeq2500 Genome Analyzer (Illumina, San Diego, CA, USA). Sequence reads were trimmed of adapter using Cutadapt [15] and aligned using either BWA or BWA MEM [16]. Genome Analysis Toolkit (GATK) v3.1 was used to perform indel realignment and Unified Genotyper was used for variant calling [17, 18]. Protein consequence and additional annotations were added using Ensembl v73 Variant Effect Predictor [19]. Variant positions were determined by reference to GenBank reference sequence NM_024675.3 according to Human Genome Variation Society (HGVS) guidelines [20]. All novel variants were validated by Sanger resequencing of germline DNA using primers from Tischkowitz et al. [21]. The following in silico prediction tools were used to assess the possible pathogenicity of missense mutations: Combined Annotation-Dependent Depletion (CADD) [22], Condel [23], SIFT [24] and PolyPhen2 [25]. CADD scores evaluate both missense and indel variants, integrating conservation measures, regulatory, transcriptional and protein effects to estimate the relative deleteriousness of the variants.

## Results

### Coverage

A total of 1996 breast cancer index cases and 1998 non-cancer controls were screened for germline mutations in the coding regions of *PALB2*. These coding regions were well covered by sequence reads in both cases and controls. The mean read depth across the entire gene for all samples was 217 (192 for cases, 242 for controls), with an average of 98.66 % of the coding regions covered by at least 20 reads (98.12 % for cases and 99.20 % for controls).

### Truncating mutations

Nineteen different truncating variants were identified in 30 individuals in this study, 26 of these variants were detected among 1996 breast cancer index cases (1.3 %) and four among 1998 controls (0.2 %), demonstrating a significant enrichment in cases (*p* = 0.0001, chi-squared test; odds ratio (OR) 6.58, 95 % confidence interval (CI) 2.3–18.9) (Table 1). Five previously reported variants were detected recurrently (c.196C > T (p.Gln66*) and c.758dupT (p.Ser254Illefs*3) each in two cases, c.3113G > A (p.Trp1038*) in seven cases and one control, and c.3116delA (p.Asn1039Ilefs*2) and c.3362delG (p.Gly1121Valfs*3) each in one case and one control) with the remaining variants detected in single cases or controls only. Six truncating variants have not previously been reported (c.522_523delAA (p.Arg175Thrfs*9), c.577dupA (p.Thr193Asnfs*2), c.693dupA (p.Gly232Argfs*3), c.860dupT (p.Ser288Lysfs*15), c.1947_1966dup (p.Pro656Glnfs*11) and c.2966_2967insCAACAAGT (p.Glu990Asnfs*3)). Novel variant p.Glu990Asnfs*3 was detected in a control only.

**Table 1:** Truncating variants

Exon	Nucleotide change^a^	Predicted protein change^a^	dbSNP ID	First reported	HAPS cases (n = 997)^b^	FCC-ViP cases (n = 999)^b^	Controls (n = 1998)^b^	CADD scaled C score
3	c.172_175delTTGT	p.Gln60Argfs*7	.	Jones (2009)	-	1	-	19.0
3	c.196C > T	p.Gln66*	rs180177083	Casadei (2011) [29]	2	-	-	35.0
4	c.522_523delAA	p.Arg175Thrfs*9	.	-	-	1	-	23.2
4	c.577dupA	p.Thr193Asnfs*2	.	-	1	-	-	11.6
4	c.693dupA	p.Gly232Argfs*3	.	-	1	-	-	11.2
4	c.758dupT	p.Ser254Ilefs*3	.	Zheng (2012) [40]	1	1	-	16.2
4	c.860dupT	p.Ser288Lysfs*15	.	-	1	-	-	17.6
5	c.1947dupA	p.Glu650Argfs*13	.	Teo (2013) [8]	-	1	-	24.1
5	c.1947_1966dup	p.Pro656Glnfs*11	.	-	1	-	-	13.0
5	c.2386G > T	p.Gly796*	rs180177112	Rahman (2007) [3]	-	1	-	32.0
5	c.2391delA	p.Gln797Hisfs*54	.	Wong-Brown (2013)	1	-	-	23.5
9	c.2966_2967insCAACAAGT	p.Glu990Asnfs*3	.	-	-	-	1	20.7
9	c.2982dupT	p.Ala995Cysfs*16	rs180177127	Rahman (2007) [3]	1	-	-	31.0
10	c.3113G > A	p.Trp1038*^c^	rs180177132	Rahman (2007) [3]	2	5	1	42.0
11	c.3116delA	p.Asn1039Ilefs*2	rs180177133	Reid (2007) [2]; Rahman (2007) [3]	-	1	1	40.0
12	c.3256C > T	p.Arg1086*	.	Jones (2009)	-	1	-	40.0
13	c.3362delG	p.Gly1121Valfs*3	.	Blanco (2013) [27]	-	1	1	22.1
13	c.3507_3508del	p.His1170Phefs*19	.	Antoniou (2014) [5]	1	-	-	40.0
13	c.3549C > G	p.Tyr1183*	rs118203998	Reid (2007) [2]	1	-	-	37.0

*HAPS* Hunter Area Pathology Service, *FCC* Familial Cancer Centre, *ViP* Variants in Practice

^a^Variant positions are reported in reference to NCBI RefSeq NM_024675.3 (mRNA) and NP_078951.2 (protein)

^b^Number of individuals carrying the variant

^c^c.3113G > A produces three different PALB2 mRNA sequences: complete deletion of exon 10 (117 bp); use of an alternative splice site within exon 10, and deletion of 31 bp; and an immediate stop at codon 1038 [29]

The personal and family history information for carriers of the *PALB2* truncating variants are given in Table 2 and Additional file 2. As expected, the cases generally have a strong family history of cancer, especially breast cancer. In the controls, four individuals were identified with truncating variants. One individual had a maternal aunt diagnosed with breast cancer at under 40 years of age, and her mother, father and brother all had cancer although not of the breast. The mothers of both of the other individuals had a breast cancer diagnosis aged over 70 years of age, and one of these individuals also had two second-degree relatives with breast cancer. The final and youngest carrier (aged 48) did not report any breast cancer in her family. Thus, 3/4 carriers have some family history of breast cancer.

**Table 2:** Family history of carriers of truncating variants

Case	Variant^a^	Diagnosis	Family history first-degree relatives	Family history other relatives
HAPS-102285	p.Gln66*	Breast 41	NA	NA
HAPS-60382	p.Gln66*	Breast 43	Mother breast 61; father prostate 65	Paternal: 2 x 2^nd^ cousins breast 42, 37; 3 x great aunt breast 42, 47,58; grandfather prostate 71. Maternal: grandmother other 60
HAPS-90978	p.Thr193Asnfs*2	Bilateral breast 61	Sister breast 60s; sister breast 60s	
HAPS-90809	p.Gly232Argfs*3	Bilateral breast 41, 47	Father lung 68	Cousin breast 45, maternal grandmother bowel 60
HAPS-120272	p.Ser254Ilefs*3	Breast 35, ovarian 58	2 x Sisters breast 40s; brother renal 52; brother HNSCC 50s; father other	Cousin breast 50s.
HAPS-102573	p.Ser288Lysfs*15	Breast 63	NA	NA
HAPS-114269	p.Pro656Glnfs*11	Breast 46, ovarian 49	Mother unknown primary 45	Maternal aunt breast 50s; grandmother breast 60s; maternal uncle prostate 70s
HAPS-120953	p.Gln797Hisfs*54	Breast 46	Father melanoma 62	half-brother NHL 64; half-brother bladder 55; maternal cousin breast 41; maternal aunt breast 55
HAPS-81242	p.Ala995Cysfs*16	Bilateral breast 46, 70	Sister breast 38; daughter breast 47; daughter cervical 24; mother breast 39	Paternal cousin breast 60s; maternal cousin other
HAPS-121549	p.Trp1038*	Breast 56	Sister breast 44	Maternal aunt breast 50s; paternal grandmother breast
HAPS-110283	p.Trp1038*	Breast 46	Mother breast 57	Maternal: aunt bowel 50; great-grandmother ovarian. Paternal: grandmother breast 85, grandfather bowel 60
HAPS-100240	p.His1170Phefs*19	Breast 48	Mother breast 68	Maternal grandmother AML 72; paternal cousin ovarian 36.
HAPS-110583	p.Tyr1183*	Breast 39	Mother breast 80; father bowel 69	Maternal aunt bilateral breast 50,70; maternal grandfather prostate 80
FCC-681-000	p.Gln60Argfs*7	Breast 47		Breast >40
FCC-2121-000	p.Gly796*	Breast 62	Lung/prostate	Prostate, other x 2
FCC-1423-000	p.Trp1038*	Breast 44	Breast >40, bowel, other	Breast >40
FCC-2104-000	p.Trp1038*	Breast 38	Breast >40, other x 2	Other
FCC-2677-000	p.Trp1038*	Cervix 55, breast 65, 67, 68, bowel 67	Breast >40, ovarian	Lung, other
FCC-3527-000	p.Trp1038*	Breast 37		Other
FCC-60-000	p.Trp1038*	Thyroid 42, breast 48, 51	Breast >40, bowel	Breast x 2 ovarian, prostate x 2, lung, other x 2
FCC-905-000	p.Asn1039Ilefs*2	Melanoma 39, breast 47	Lung, prostate, other	Breast x 3, bowel x 2, ovarian
FCC-2965-000	p.Arg1086*	Bilateral breast 44	Prostate	
FCC-317-000	p.Gly1121Valfs*3	Breast 31	Other x 2	Breast, other
FCC-1322-000	p.Glu650Argfs*13	Breast 50	Breast, other	Breast x 3, other
FCC-3397-000	p.Ser254Ilefs*3	Melanoma 53, Breast 54, 61	Breast	
FCC_2431-000	p.Arg175Thrfs*9	Breast 42	Breast, other x 3	Breast x 3, lung, other x 2
**Controls**	**Variant**	**Age enrolled**	**Family history first-degree relatives**	**Family history other relatives**
LP-12031915	p.Trp1038*	65	Mother, other 58; father lung 64; brother other	Maternal aunt breast >40
LP-13099711	p.Asn1039Ilefs*2	73	Mother breast 72	Maternal: grandmother breast >40 yrs; aunt breast
LP-13243620	p.Glu990Asnfs*3	73	Mother breast 82	NA
LP-12025195	p.Gly1121Valfs*3	48	None	Yes, no details

*NA* not applicable, *HNSCC* head and neck squamous cell carcinoma, NHL non-Hodgkin lymphoma

^a^Novel variants in italics

### Missense and synonymous variants

A large number of missense variants (n = 54) were detected in the cohort (Table 3). There was a slight enrichment for missense variants overall in cases (39.6 %) versus controls (36.1 %, *p* = 0.025, OR 1.15, 95 % CI 1.02–1.32). The four most common variants (>3 % carrier frequency) were tested for association with breast cancer. Surprisingly, the most common variant, c.1676A > G (Gln559Arg; rs152451), which is predicted by CADD, Condel, PolyPhen2 and SIFT to be benign, was significantly more common in cases (19.9 % carried at least one non-wild-type allele) than controls (16.8 % non-wild-type) with a per-allele OR of 1.24 (95 % CI 1.09–1.47, *p* = 0.002, logistic regression). There were 20 cases homozygous for this variant versus ten controls (*p* = 0.058 logistic regression, OR 2.09, 95 % CI 0.98–4.48). The overall trend for an effect had a *p* value of 0.0018 (Cochrane-Armitage trend test). The minor allele frequency was 0.105 in cases and 0.086 in controls, compared to other databases where the minor allele frequency of this variant in European populations was 0.09 (1000 Genomes), 0.096 (ExAC) and 0.09 (EVS), but showed increased frequency in African and Asian populations.

**Table 3:** Missense variants

Exon	Nucleotide change^a^	Protein change^a^	dbSNP ID	First reported	1000 G MAF^b^	NHBLI GO ESP MAF^c^	ExAC^d^	HAPS cases (n = 997)^e^	FCC-ViP cases (n = 999)^e^	Controls (n = 1998)^e^	CADD scaled C score	Condel	PolyPhen2	SIFT
1	c.11C > T	p.Pro4Leu	rs45619737	Rahman (2007) [3]	.	0.00038	0.0000999	1	1	1	12.9	Deleterious	Probably damaging	Tolerated
2	c.53A > G	p.Lys18Arg	rs138789658	Tischkowitz (2008)	0.0032	0.00523	0.0000552	-	-	1	18.0	Deleterious	Possibly damaging	Deleterious
2	c.94C > G	p.Leu32Val	rs151316635	Teo (2013) [8]	.	0.00023	0.0000184	-	1	1	16.3	Deleterious	Possibly damaging	Deleterious
3	c.194C > T	p.Pro65Leu	rs62625272	Adank (2011) [26]	.	0.00015	0.00006	-	-	1	4.6	Neutral	Benign	Tolerated
4	c.232G > A	p.Val78Ile	.	Tischkowitz (2012) [39]	.	.	0.000325		2		0.4	Neutral	Benign	Tolerated
4	c.298C > T	p.Leu100Phe	rs61756147	Wong (2011) [9]	0.0005	0.00023	0.000037	-	-	2	10.3	Neutral	Probably damaging	Tolerated
4	c.344G > T	p.Gly115Val	rs145598272	Foulkes (2007) [34]	.	0.00015	0.0000921	1	-	1	6.5	Neutral	Benign	Tolerated
4	c.353 T > C	p.Ile118Thr	.	-	.	0.00008	0.000037	-	-	1	5.4	Neutral	Benign	Tolerated
4	c.400G > A	p.Asp134Asn	rs139555085	Zheng (2011)	0.0005	0.00184	0			1	4.8	Neutral	Benign	Tolerated
4	c.508A > G	p.Arg170Gly	.	-	.	.	.	-	-	1	4.4	Neutral	Benign	Tolerated
4	c.557A > T	p.Asn186Ile	.	-	.	.	.	-	-	1	13.1	Deleterious	Probably damaging	Deleterious
4	c.571C > G	p.Pro191Ala	.	-	.	.	.	-	-	1	9.4	Neutral	Probably damaging	Tolerated
4	c.629C > T	p.Pro210Leu	rs57605939	Rahman (2007) [3]	0.0174	0.02216	0.0000736	1	1	2	10.6	Deleterious	Probably damaging	Tolerated
4	c.899C > T	p.Thr300Ile	.	Ding (2011)	.	.	0	1	-	-	15.1	Deleterious	Probably damaging	Tolerated
4	c.656A > G	p.Asp219Gly	rs45594034	Rahman (2007) [3]	.	0.00015	0.000221		1		1.8	Neutral	Benign	Tolerated
4	c.740C > G	p.Thr247Arg	.	.	.	.	.		1		15.5	Deleterious	Probably damaging	Tolerated
4	c.925A > G	p.Ile309Val	rs3809683	Rahman (2007) [3]	0.0087	0.00970	0.0000184	4 (1)	-	-	0.5	Neutral	Benign	Tolerated
4	c.1010 T > C	p.Leu337Ser	rs45494092	Rahman (2007) [3]	0.0133	0.01424	0.0197	44 (1)	44	93 (1)	8.9	Deleterious	Probably damaging	Tolerated
4	c.1085 T > C	p.Leu362Pro	.	-	.	.	.	1	-	-	14.4	Deleterious	Probably damaging	Tolerated
4	c.1145G > T	p.Ser382Ile	.	Tischkowitz (2012) [39]	.	.	0.0000184	-	1	-	15.2	Deleterious	Possibly_damaging	Deleterious
4	c.1189A > T	p.Thr397Ser	.	Rahman (2007) [3]	.	0.00008	0.0000184	-	1	1	22.9	Deleterious	Possibly damaging	Deleterious
4	c.1250C > A	p.Ser417Tyr	rs45510998	Rahman (2007)	.	.	0.000203	-	-	1	20.6	Deleterious	Probably damaging	Deleterious
4	c.1478C > T	p.Pro493Leu	.	-	.	.	0.0000184	-	-	1	12.4	Neutral	Benign	Tolerated
4	c.1492G > T	p.Asp498Tyr	rs75023630	Phuah (2013) [38]	0.0014	.	0	-	-	1	16.2	Deleterious	Benign	Deleterious
4	c.1544A > G	p.Lys515Arg	.	Tischkowitz (2012) [39]	.	.	0.000037	-	1	-	16.2	Deleterious	Possibly damaging	Tolerated
4	c.1610C > T	p.Ser537Leu	rs142103232	-	.	0.00015	0.000166	-	1	-	11.2	Neutral	Possibly damaging	Tolerated
4	c.1676A > G	p.Gln559Arg	rs152451	Rahman (2007) [3]	0.1465	0.13483	0.0961	216 (9)	182 (11)	335 (10)	0.0	Neutral	Benign	Tolerated
5	c.1699C > T	p.His567Tyr	.	Tischkowitz (2012) [39]	.	0.00008	0.000094	-	-	1	0.9	Neutral	Benign	Tolerated
5	c.1931G > A	p.Gly644Glu	.	-	.	.	.	-	-	1	16.0	Deleterious	Probably damaging	Deleterious
5	c.2014G > C	p.Glu672Gln	rs45532440	Rahman (2007) [3]	0.0142	0.02324	0.0278	64 (1)	68 (4)	123 (1)	11.4	Neutral	Possibly damaging	Tolerated
5	c.2106A > G	p.Ile702Met	.	-	.	.	0.0000184	1	-	-	11.1	Neutral	Probably damaging	Tolerated
5	c.2135C > T	p.Ala712Val	rs141458731	Dansonka-Meiszkowska (2010)	0.0014	0.00062	0.00039	-	-	1	12.2	Neutral	Benign	Tolerated
5	c.2200A > T	p.Thr734Ser	rs45543843	Rahman (2007) [3]	.	.	0.0000368	-	-	3	25.3	Deleterious	Possibly damaging	Deleterious
5	c.2228A > G	p.Tyr743Cys	rs141749524	-	0.0009	.	0.0000184	1	-	-	8.5	Neutral	Benign	Tolerated
5	c.2289G > C	p.Leu763Phe	.	Phuah (2013) [38]	.	.	0	2	-	-	15.5	Neutral	Probably damaging	Tolerated
5	c.2360C > T	p.Thr787Ile	rs201042302	-	0.0005	.	0	-	1	-	9.7	Neutral	Probably damaging	Tolerated
5	c.2417C > T	p.Pro806Leu	rs45464991	Rahman (2007) [3]	.	0.00008	0.000037	-	1	1	0.5	Neutral	Benign	Tolerated
7	c.2590C > T	p.Pro864Ser	rs45568339	Rahman (2007) [3]	0.0018	0.00239	0.00396	6	10	19	12.0	Neutral	Benign	Tolerated
7	c.2606C > G	p.Ser869Cys	.	.	.	.	0.0000184		1		19.9	Deleterious	Probably damaging	Deleterious
7	c.2674G > A	p.Glu892Lys	rs45476495	Rahman (2007) [3]	.	0.00008	0.000092	1	2	-	20.8	Deleterious	Possibly damaging	Deleterious
8	c.2755G > A	p.Val919Ile	.	-	.	.	.	-	-	1	15.7	Neutral	Benign	Tolerated
8	c.2794G > A	p.Val932Met	rs45624036	Rahman (2007) [3]	0.0009	0.00431	0.00869	15	8	23	18.3	Deleterious	Probably damaging	Tolerated
8	c.2816 T > G	p.Leu939Trp	rs45478192	Rahman (2007) [3]	0.0009	0.00154	0.0015	2	3 (1)	8	20.9	Deleterious	Probably damaging	Deleterious
9	c.2993G > A	p.Gly998Glu	rs45551636	Rahman (2007) [3]	0.0105	0.01785	0.0213	42	46 (2)	92 (1)	22.7	Deleterious	Probably damaging	Deleterious
10	c.3054G > C	p.Glu1018Asp	rs183489969	Tischkowitz (2012) [39]	0.0009	.	0	1	-	1	16.4	Deleterious	Possibly damaging	Deleterious
10	c.3106G > C	p.Val1036Leu	.	-	.	.	0.000037	-	1	-	13.3	Neutral	Benign	Tolerated
11	c.3128G > C	p.Gly1043Ala	.	Hellebrand (2011) [36]	0.0009	.	0.000037	1	-	-	20.9	Deleterious	Probably damaging	Deleterious
11	c.3146 T > C	p.Met1049Thr	rs138273800	-	.	0.00008	.	-	1	-	18.0	Deleterious	Probably damaging	Deleterious
12	c.3307G > A	p.Val1103Met	rs201657283	Casadei (2011) [29]	.	0.00015	0.0000184	-	1	-	13.0	Neutral	Benign	Tolerated
13	c.3366C > A	p.Asp1122Glu	.	.	.	.	.	-		1	16.0	Neutral	Possibly damaging	Tolerated
13	c.3367G > A	p.Val1123Met			.	.	.	-	1	-	18.7	Deleterious	Probably damaging	Deleterious
13	c.3428 T > A	p.Leu1143His	rs62625284	Balia (2010)	.	0.00008	0.00031	2	-	-	20.0	Deleterious	Possibly damaging	Tolerated
13	c.3448C > T	p.Leu1150Phe			.	.	.	-	1	-	15.5	Deleterious	Possibly damaging	Deleterious
13	c.3449 T > G	p.Leu1150Arg	rs45566737		.	.	0.0000368	-	1	-	18.2	Deleterious	Possibly damaging	Deleterious

*HAPS* Hunter Area Pathology Service, *FCC* Familial Cancer Centre, *ViP* Variants in Practice

^a^Variant positions are reported in reference to NCBI RefSeq NM_024675.3 (mRNA) and NP_078951.2 (protein)

^b^Minor allele frequency (MAF) reported in the 1000 Genomes (1000 G) cohort Phase 1

^c^MAF reported in the Exome Variant Server, NHLBI GO Exome Sequencing Project (ESP) [46] (data release ESP6500SI-V2)

^d^MAF reported in ExAC [45] from non-Finnish Europeans, excluding individuals in the database who were part of The Cancer Genome Atlas and therefore known to have had cancer

^e^Number of individuals carrying the variant. Where applicable, the number of homozygous carriers is indicated in parentheses

Considering only those rare variants present in fewer than five carriers among 3994 cases and controls (approximately 0.1 %), a similar number of missense variants were detected in both groups (40 in cases (2 %), 28 in controls (1.4 %)), which does not suggest any association of rare missense variants with risk. There was also no significant enrichment in cases when limited to rare variants that were predicted to be deleterious by any of Condel, SIFT or Polyphen2 (28/1996 cases, 18/1998 controls) or with a CADD score of >10 (29/1996 cases, 20/1998 controls).

We detected 23 synonymous variants (Table 4). Neither the most common alone (c.3300 T > G) nor all together were significantly enriched in cases or controls.

**Table 4:** Synonymous variants

Exon	Nucleotide change^a^	Protein change^a^	dbSNP ID	First reported	1000 G MAF^b^	NHBLI GO ESP MAF^c^	ExAC^d^	HAPS cases (n = 997)^e^	FCC-ViP cases (n = 999)^d^	Controls (n = 1998)^e^	CADD scaled C score
1	c.12 T > C	p.(=)	rs145291423	-	.	.	0.00012	-	1	1	11.2
4	c.768C > T	p.(=)	rs45487491		.	.	0.000037		1		0.3
4	c.1188C > T	p.(=)	.	-	.	.	0.00003	-	-	1	7.6
4	c.1194G > A	p.(=)	rs61755173	Rahman (2007) [3]	0.0009	0.00154	0.00114	3	2	8	6.4
4	c.1194G > T	p.(=)	.	-	.	.	.	1	-	-	6.1
4	c.1242A > C	p.(=)	.	-	.	.	.	-	-	1	7.7
4	c.1431C > T	p.(=)	.	Teo (2013)	.	.	0	2	-	-	0.0
4	c.1470C > T	p.(=)	rs45612837	Rahman (2007) [3]	0.0005	0.00015	0.000405	1	-	2	5.8
4	c.1572A > G	p.(=)	rs45472400	Rahman (2007) [3]	0.0032	0.00339	0.0041	12	12	27	5.6
4	c.1623G > A	p.(=)	.	-	.	.	0	-	1	2	4.2
5	c.2067G > A	p.(=)	.	Phuah (2013) [38]	.	0.00015	0.000018	1	-	-	4.1
5	c.2082A > G	p.(=)	rs150569240	-	.	.	0.000055	-	-	1	5.8
5	c.2091C > A	p.(=)	.	-	.	.	0	-	-	1	4.7
5	c.2244A > G	p.(=)	.	-	.	.	0.000037	-	-	1	5.6
5	c.2328C > T	p.(=)	rs45508997	-	.	0.00008	0	1	-	-	4.0
5	c.2337A > C	p.(=)	.	-	.	.	.	-	1	-	5.2
5	c.2379C > T	p.(=)	.	-	.	0.00008	0.000111	1	1	-	0.1
5	c.2478C > T	p.(=)	.	-	.	.	.	-	-	1	4.2
5	c.2484C > T	p.(=)	.	-	.	.	.	-	-	1	6.0
7	c.2742C > T	p.(=)	rs115759702	-	0.0018	0.00146	0.00003	2	-	-	0.7
12	c.3294G > A	p.(=)	.	-	.	.	.	-	-	1	9.9
12	c.3300 T > G	p.(=)	rs45516100	Rahman (2007) [3]	0.0183	0.02801	0.0278	64 (1)	68 (4)	123 (1)	7.9
13	c.3495G > A	p.(=)	rs45439097	Bogdanova (2010)	.	0.00108	0.000994	4	7	9	8.6

*HAPS* Hunter Area Pathology Service, *FCC* Familial Cancer Centre, *ViP* Variants in Practice

^a^Variant positions are reported in reference to NCBI RefSeq NM_024675.3 (mRNA) and NP_078951.2 (protein)

^b^Minor allele frequency (MAF) reported in the 1000 Genomes (1000 G) cohort Phase 1

^c^MAF reported in the Exome Variant Server, NHLBI GO Exome Sequencing Project (ESP) [46] (data release ESP6500SI-V2)

^d^MAF reported in ExAC [45] from non-Finnish Europeans, excluding individuals in the database who were part of The Cancer Genome Atlas and therefore known to have had cancer

^e^Number of individuals carrying the variant. Where applicable, the number of homozygous carriers is indicated in parentheses

## Discussion

This study screened Australian individuals with breast cancer who had been referred to a Familial Cancer Centre for genetic testing and in whom no pathogenic *BRCA1* and *BRCA2* variant could be identified. The frequency of *PALB2* truncating variants in this cohort (1.1 %) is similar to other studies analysing high-risk breast cancer individuals (0.64–3.4 %, 1.35 % overall [3, 6–9, 26–41]) or triple-negative breast cancer (0.9–2.5 % [10, 42, 43]) but is the largest to include an analysis of the full gene in both cases and controls. However, we would not be able to detect any large deletions or rearrangements. The low frequency of truncating variants in controls supports *PALB2* as a high-penetrance breast cancer predisposing gene. The diversity of truncating mutations identified, comprising 16 different variants in eight of the 13 exons including five novel variants, highlights the need for full gene screening, not just the most common variant c.3113G > A (rs180177132). These data will enable evidence-based clinical guidelines to include full *PALB2* screening if previously they had advised testing limited to the specific common variant only.

The prevalence of truncating variants in cancer-free controls was 0.15 % in the LifePool cohort. These individuals were ascertained from women attending population-based mammographic screening, which in Australia is targeted towards women over 50, although some younger women are included. Thus, this volunteer cohort may not be entirely representative of the general population, although all were cancer-free at the time of analysis. Nonetheless, the frequencies of missense and synonymous variants are consistent with those reported in large databases such as 1000 Genomes [44], Exome Aggregation Consortium [45] and Exome Variant Server [46].

We did not observe any significant enrichment in missense mutations overall, although the frequency was slightly higher in the cases when only rare, deleterious mutations were considered. The contribution of rarer variants to breast cancer risk will need to be evaluated in larger case–control cohorts. Surprisingly, the common variant c.1676A > G (Gln559Arg; rs152451) was significantly enriched in cases versus controls, although with only a modest odds ratio (1.24). There was a trend towards homozygous carriers of this variant being enriched in cases versus controls with an OR of 2.08. This variant was shown to be associated with an increased breast cancer risk in multiple-case breast cancer families in Chile compared to population controls [47] with an OR of 2.0 when at least three family members were breast or ovarian cancer-affected. No association was found for individuals diagnosed at a young age (<50) and with no affected relatives. In a small Malaysian case–control study, there was a trend towards enrichment for carriers of the variant in non-familial breast cancer cases (286/871, 33 %) versus controls (70/257, 27 %, OR 1.3 [38]), however, cases and controls were not well matched for ethnicity, with an excess of Indian and Malay women over Chinese in the controls compared to cases. Larger numbers of cases and controls will be required to confirm whether the association of rs152451 with breast cancer is a robust finding. In addition, the wide variation in the frequency of the minor allele in different populations means that cases and controls will have to be carefully matched for ethnicity. This variant is not located in a known protein domain and was consistently found to have predicted benign effects on protein function by all algorithms tested. However, this base change is only 9 bp away from the exon 4 splice donor site and Human Splicing Finder (v3) found that rs152451 could alter an exonic splicing enhancer motif [48], offering a potential mechanism for how this variant could affect *PALB2* function. It should be noted that such a prediction was relatively common for the variants we detected in *PALB2* (35/77 missense or nonsynonymous variants had a similar prediction from at least three algorithms) and any effect would need to be confirmed by an RNA-based assay.

There has been only one study to date that has examined the likely functional effect of missense variants in *PALB2*, which examined p.Leu939Trp, p.Leu1143Pro and p.Thr1030Ile [49]. The first two variants had subtle but significant effects on homologous recombination repair: p.Leu1143Pro in particular showed decreased repair capacity and binding to BRCA2 and RAD51C. PALB2 p.Thr1030Ile was unstable, leading to decreased protein levels and this was assumed to impair homologous recombination repair. However, it should be noted that these functional assays were performed by overexpression of a retroviral transgene in a null cell line and may not reflect the heterozygote situation. In our study, p.Leu939Trp was not enriched in cases (four in cases, eight in controls), p.Leu1143Pro was only seen in two cases and no controls, while p.Thr1030Ile was not observed in either cases or controls.

## Conclusions

Our data strongly support *PALB2* as a breast cancer predisposition gene when considering truncating mutations. We did not see any excess in missense mutations in cancer cases overall, although there may be individual variants that are associated with risk at low penetrance. We advise extreme caution in attributing risk to missense *PALB2* mutations when determining clinical management.

## Additional files

Additional file 1:
**Cohort information (Table).** (DOCX 48 kb) Additional file 2:
***PALB2*** **truncating variant carrier family pedigrees (Figure).** (PDF 1036 kb)

## Abbreviations

CI: confidence interval
FCC: Familial Cancer Centre
HAPS: Hunter Area Pathology Service
OR: odds ratio
ViP: Variants in Practice
