# Supplementary material for: Prevalence of PALB2 mutations in Australian familial breast cancer cases and controls
Source: Breast Cancer Res. 2015 Aug 19;17(1):111. doi: 10.1186/s13058-015-0627-7 (PMC4539664; doi:10.1186/s13058-015-0627-7)
Supplement: Additional file 1: — Cohort information (Table). (DOCX 48 kb) [file 13058_2015_627_MOESM1_ESM.docx]

**Supplementary Table 1. Cohort information**

|  | **FCC** | **HAPS** |
| --- | --- | --- |
| **Year of diagnosis** | **1997-2014** | **2008-2012** |
| **Average age (standard deviation)** | **45.3 (10.3)** | **53.8 (12.2)** |
| **<50** | **662** | **381** |
| **<50 with family history of breast/ovarian cancer in 1^st^ and/or 2^nd^ degree relatives** | **462 (70% of <50s)** | **Not known** |
| ≥ **50 with family history or breast/ovarian cancer in 1^st^ and/or 2^nd^ degree relatives** | **286 (90% of** ≥ **50s)** | **Not known** |
