# Supplementary material for: Prevalence of PALB2 mutations in Australian familial breast cancer cases and controls
Source: Breast Cancer Res. 2015 Aug 19;17(1):111. doi: 10.1186/s13058-015-0627-7 (PMC4539664; doi:10.1186/s13058-015-0627-7)

FCC-60-00 p.Trp1038*

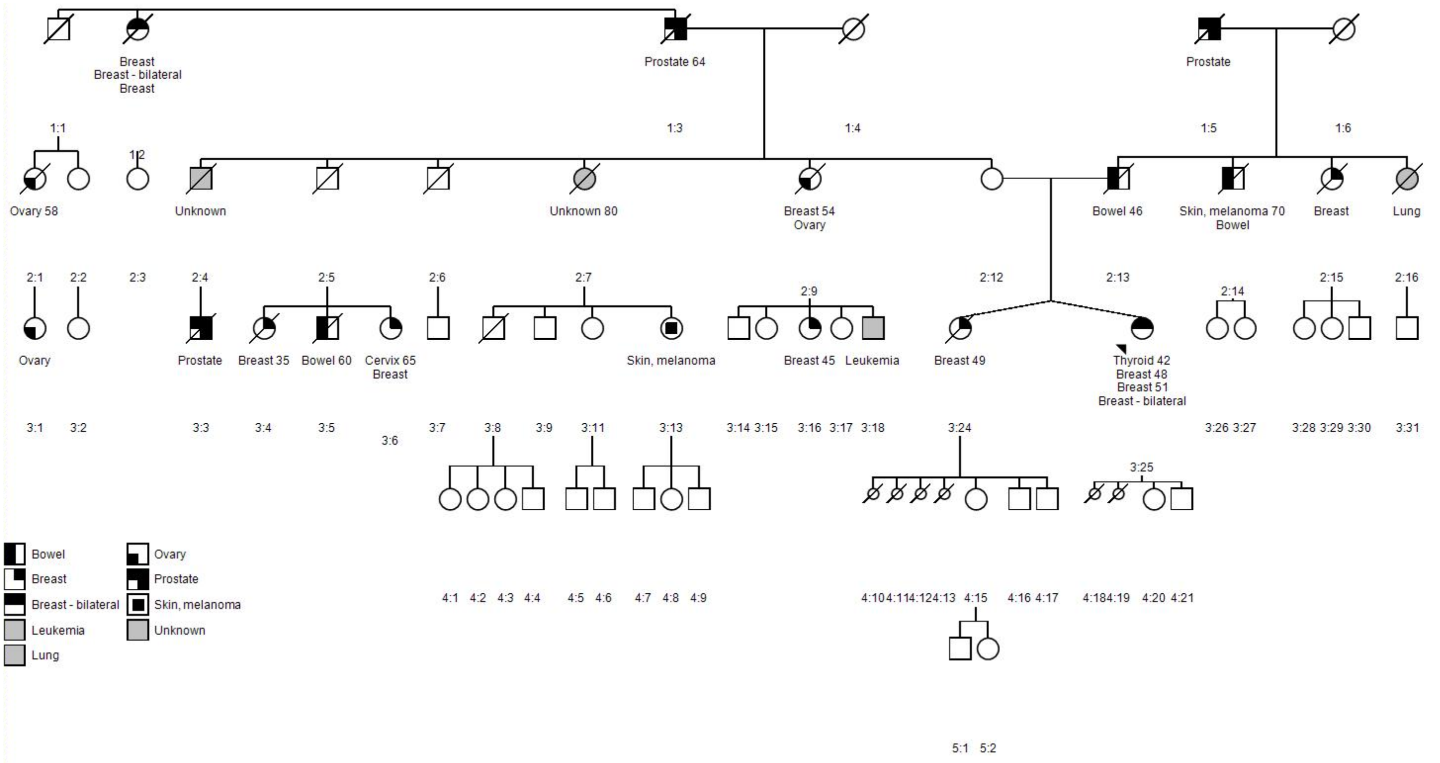

FCC-317-000
p.Gly1121Valfs*3

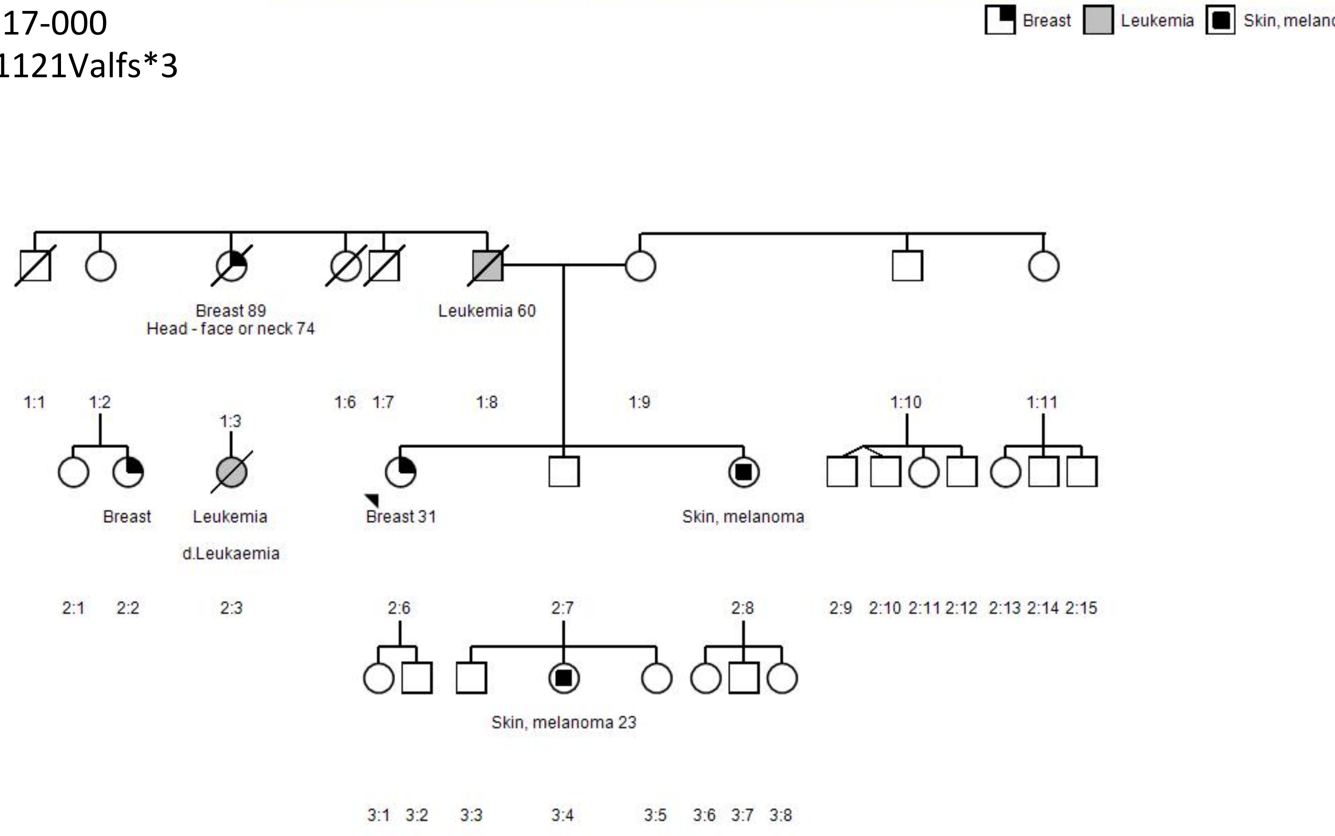

FCC-681-000
p.Gln60Argfs*7

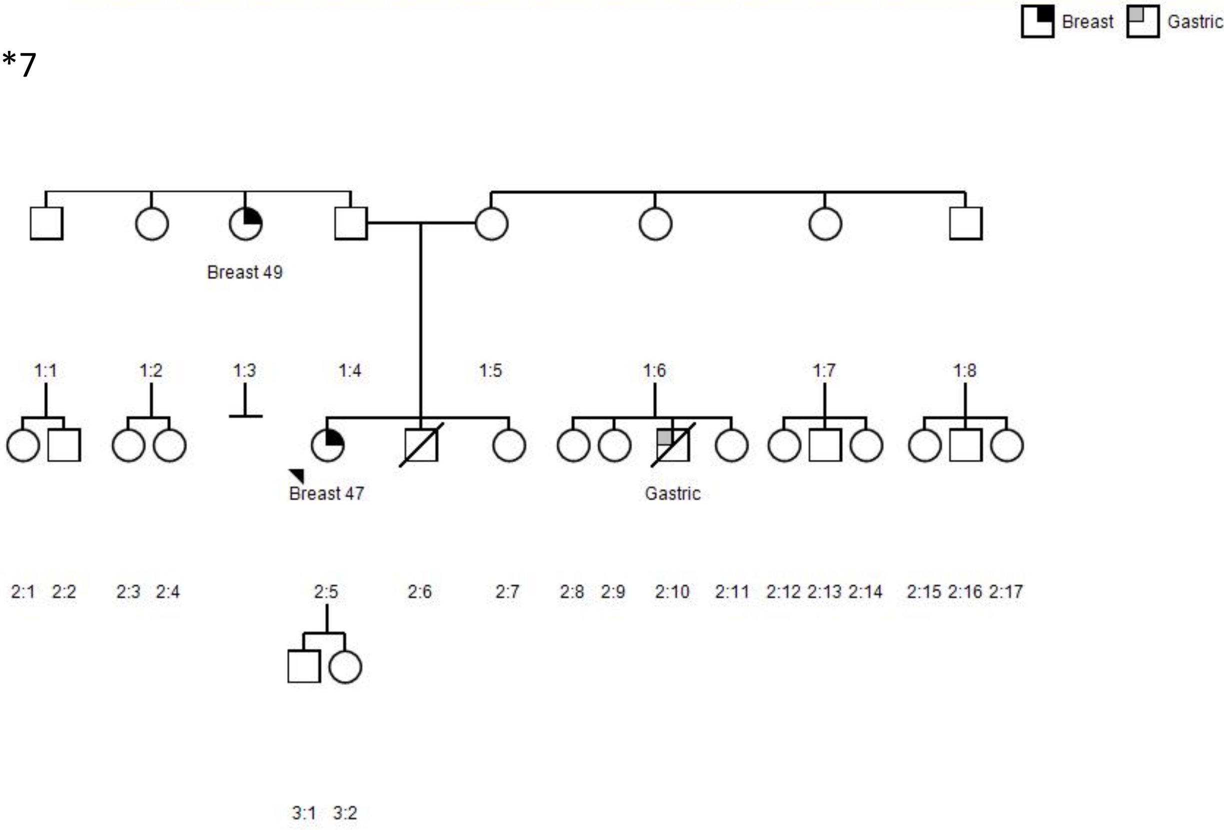

FCC-905-000
p.Asn1039Ilefs*2

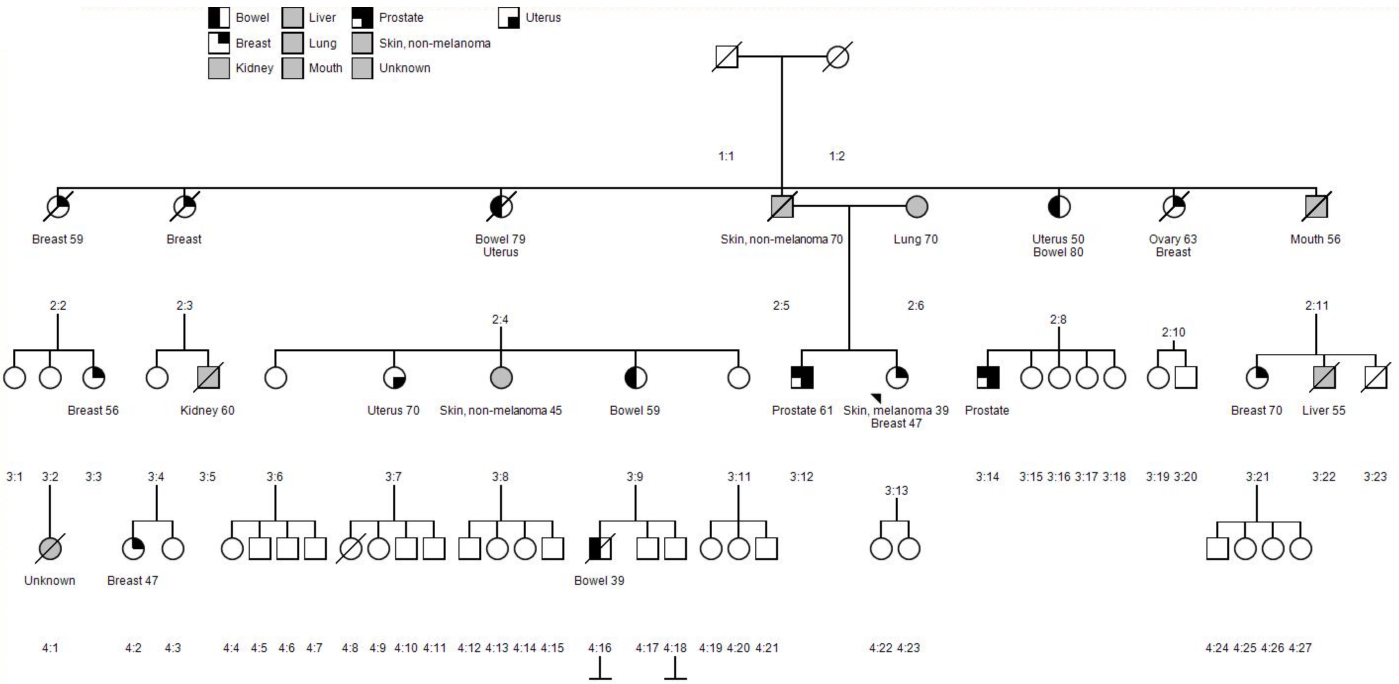

FCC-1322-000
p.Glu650Argfs*13

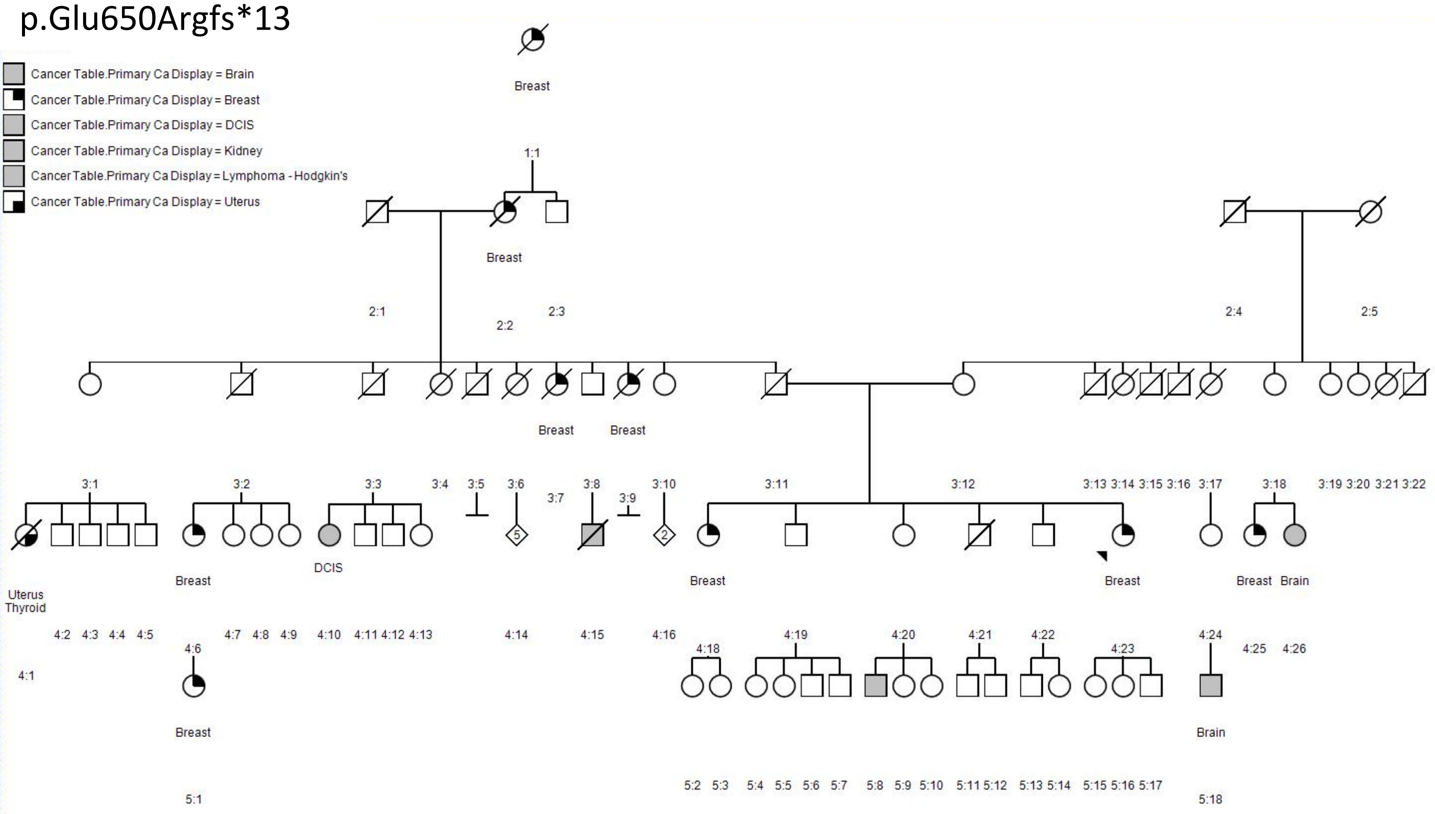

FCC-1423-000
p.Trp1038*

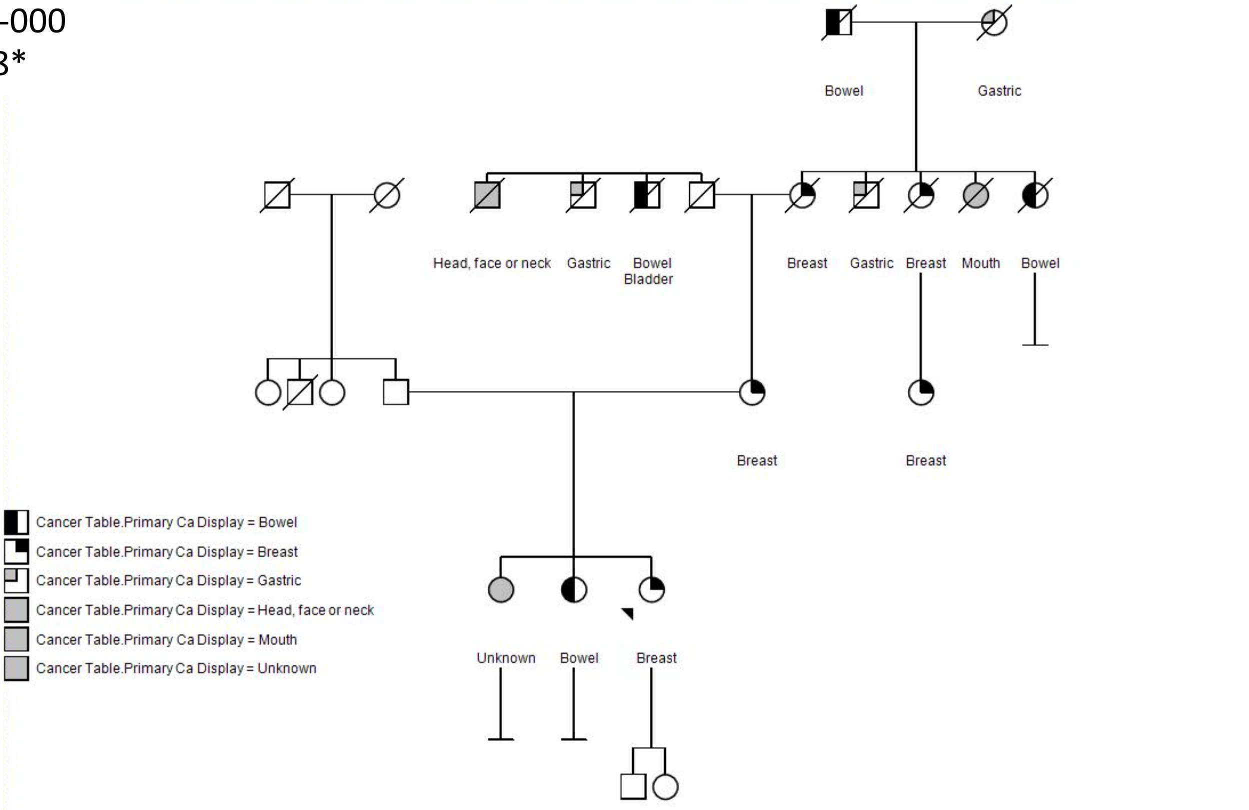

FCC-2104-000
p.Trp1038*

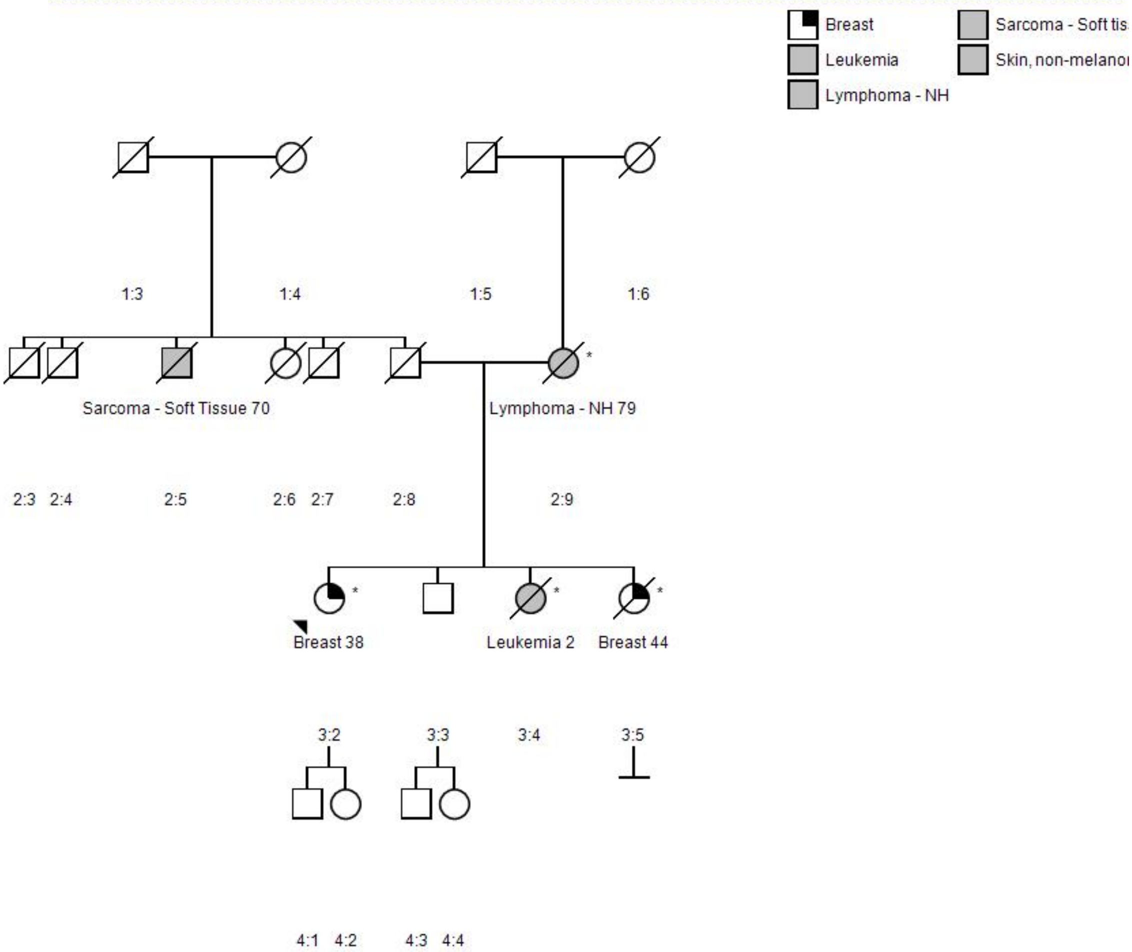

FCC-2121-000
p.Gly796*

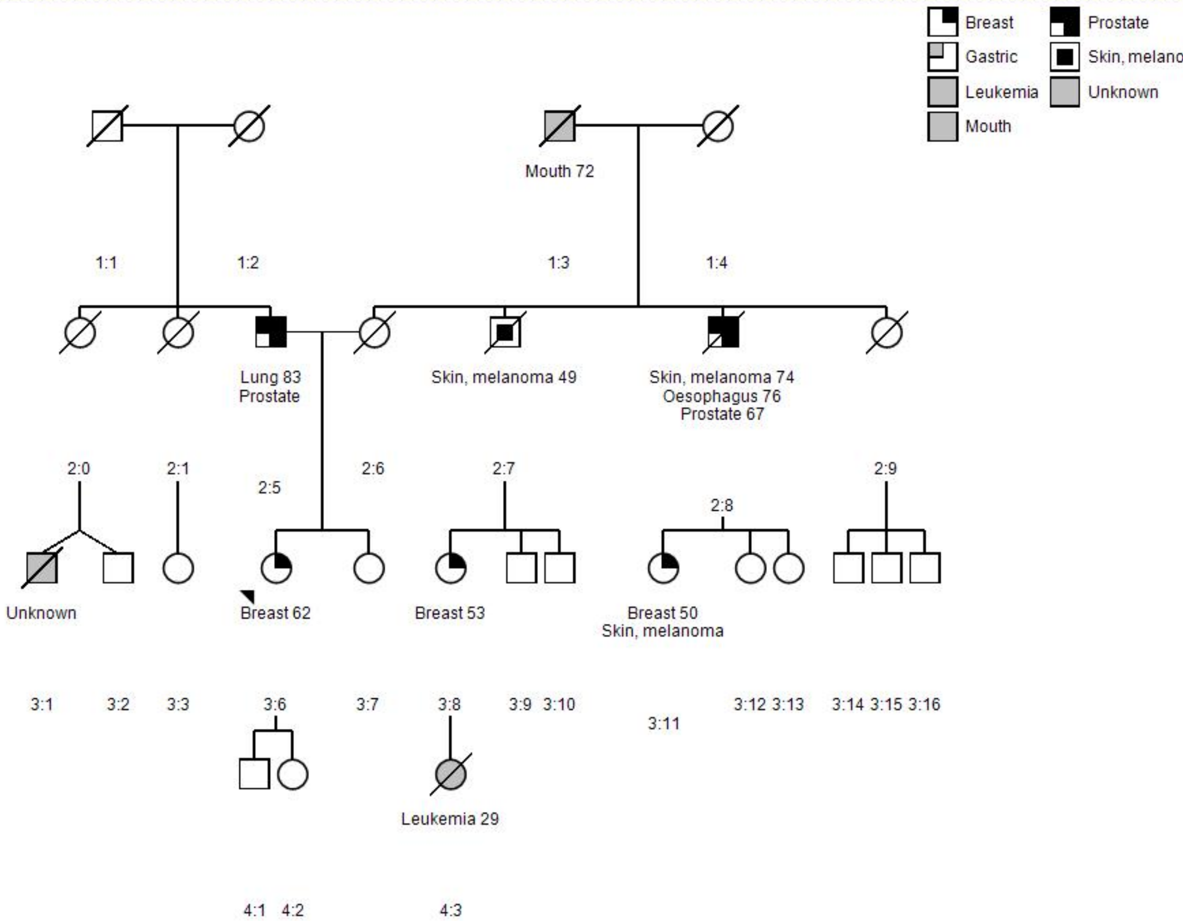

FCC-2431-000
p.Arg175Thrfs*9

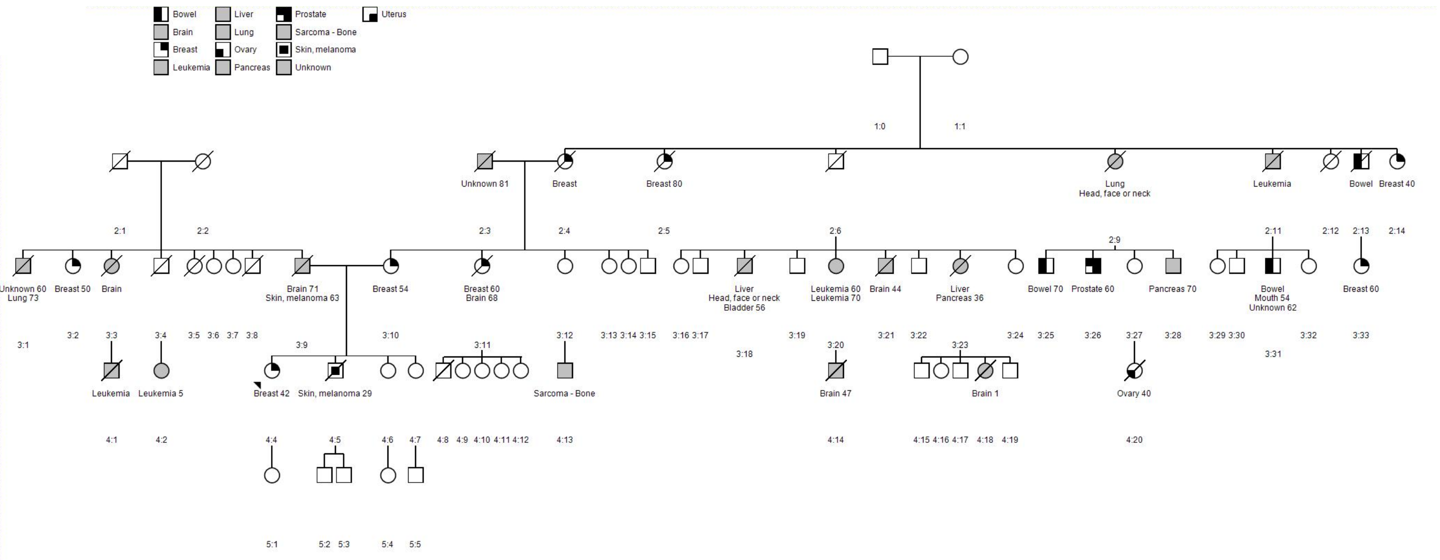

FCC-2677-000
p.Trp1038*

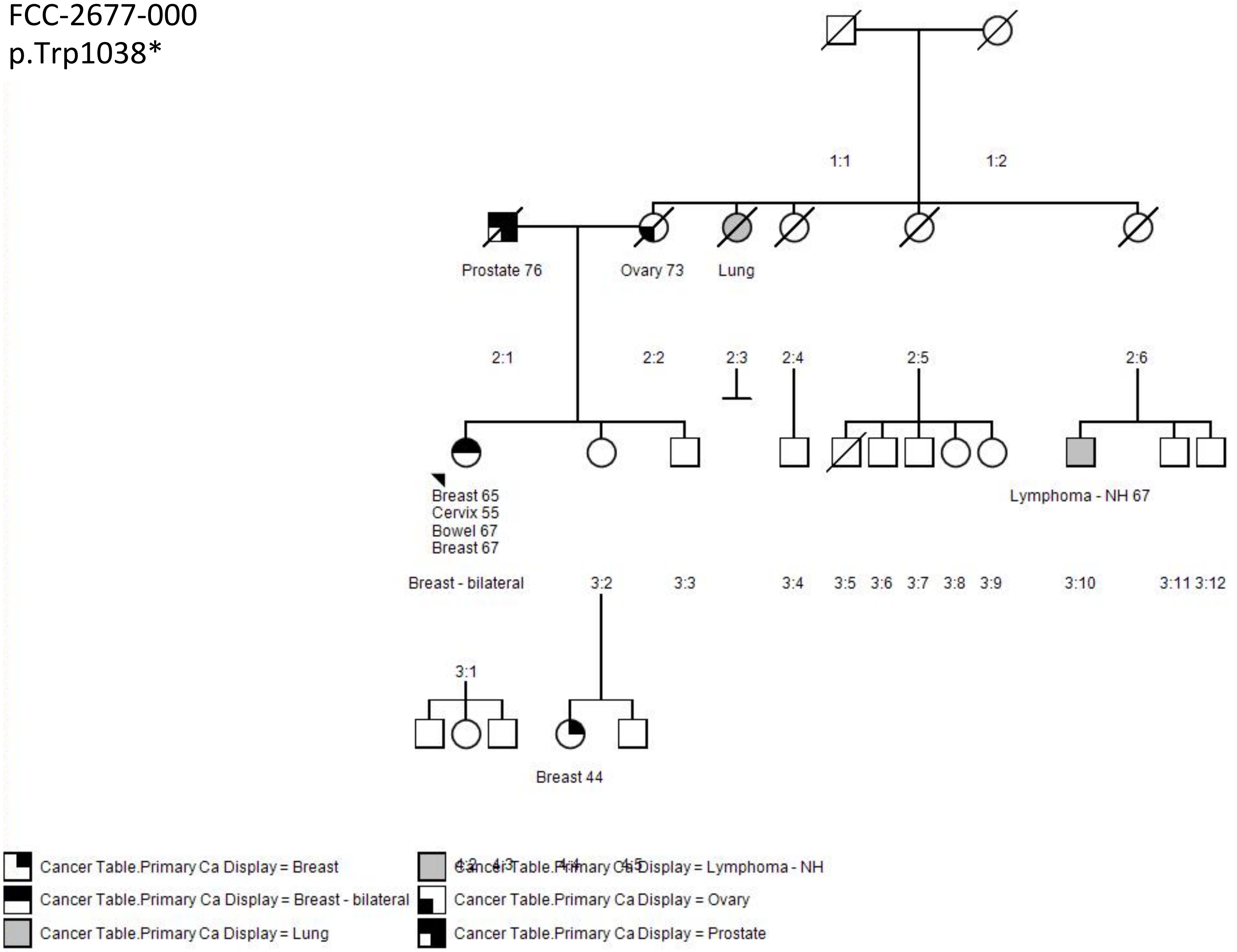

FCC-3397-000
p.Ser254Ilefs*3

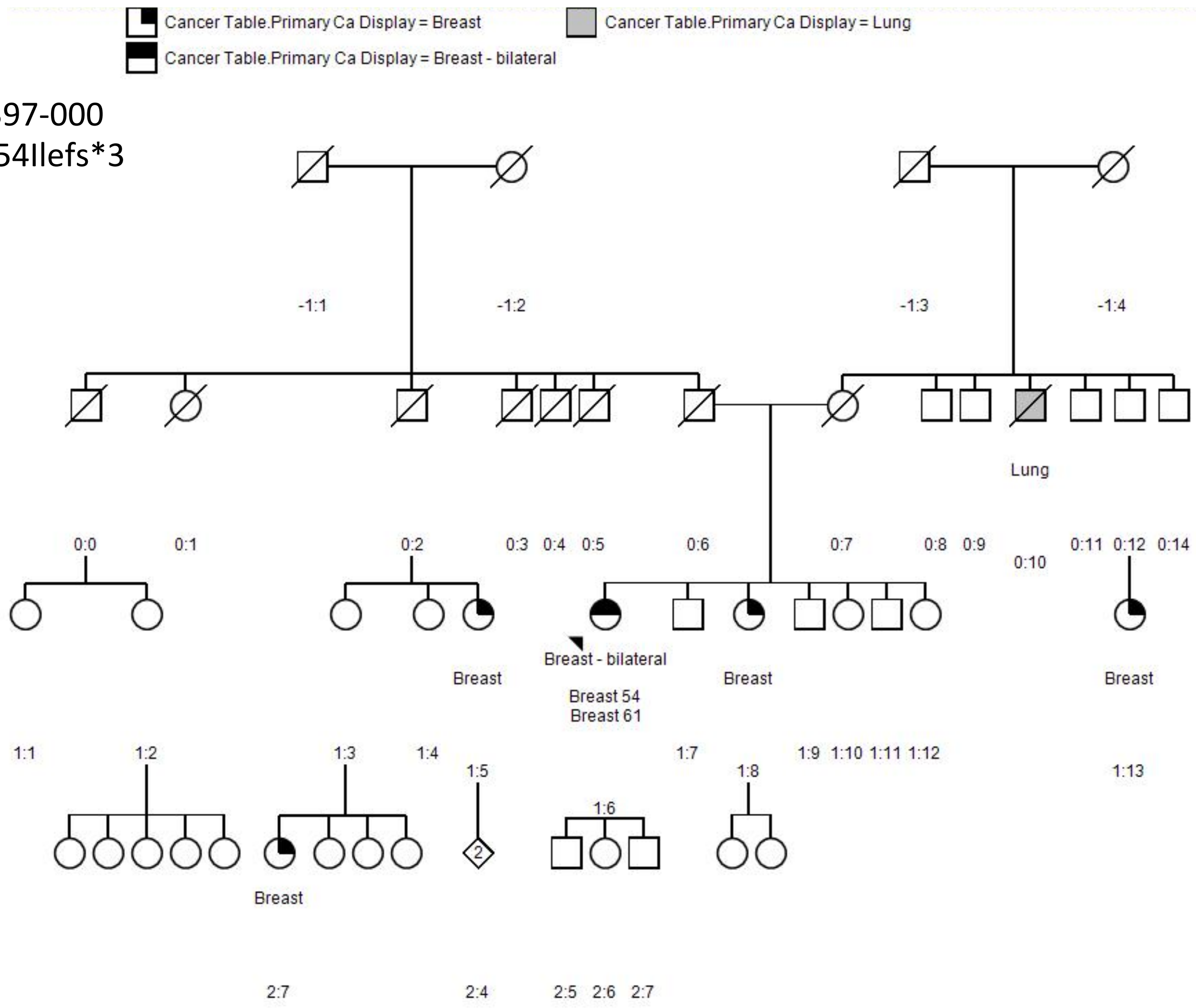

FCC-3527-000
p.Trp1038*

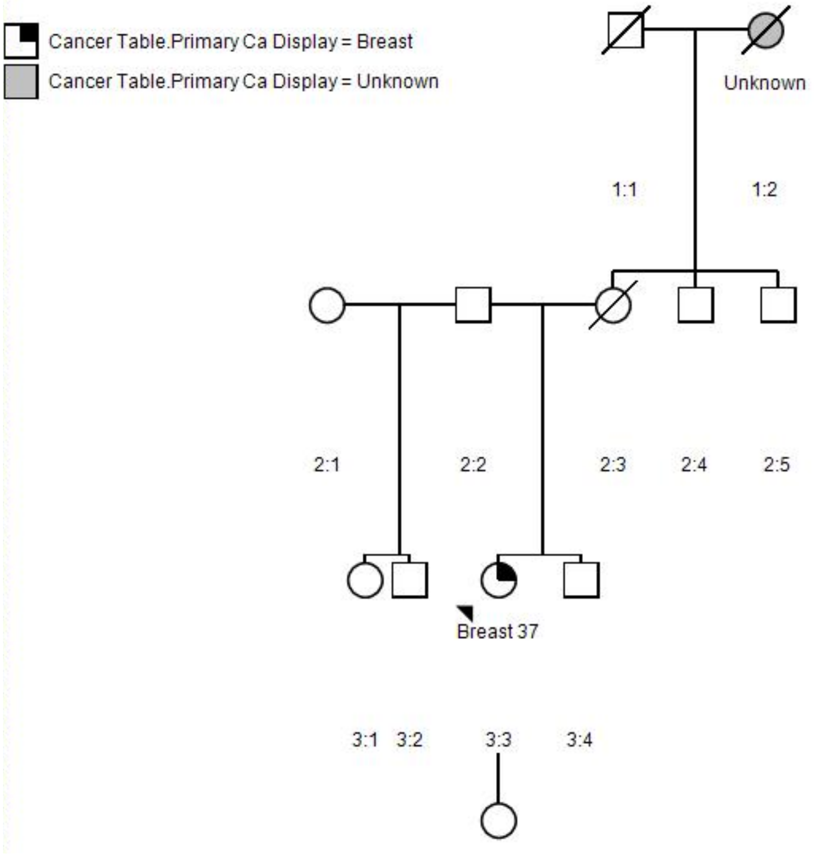

FCC-2965-000
p.Arg1086*

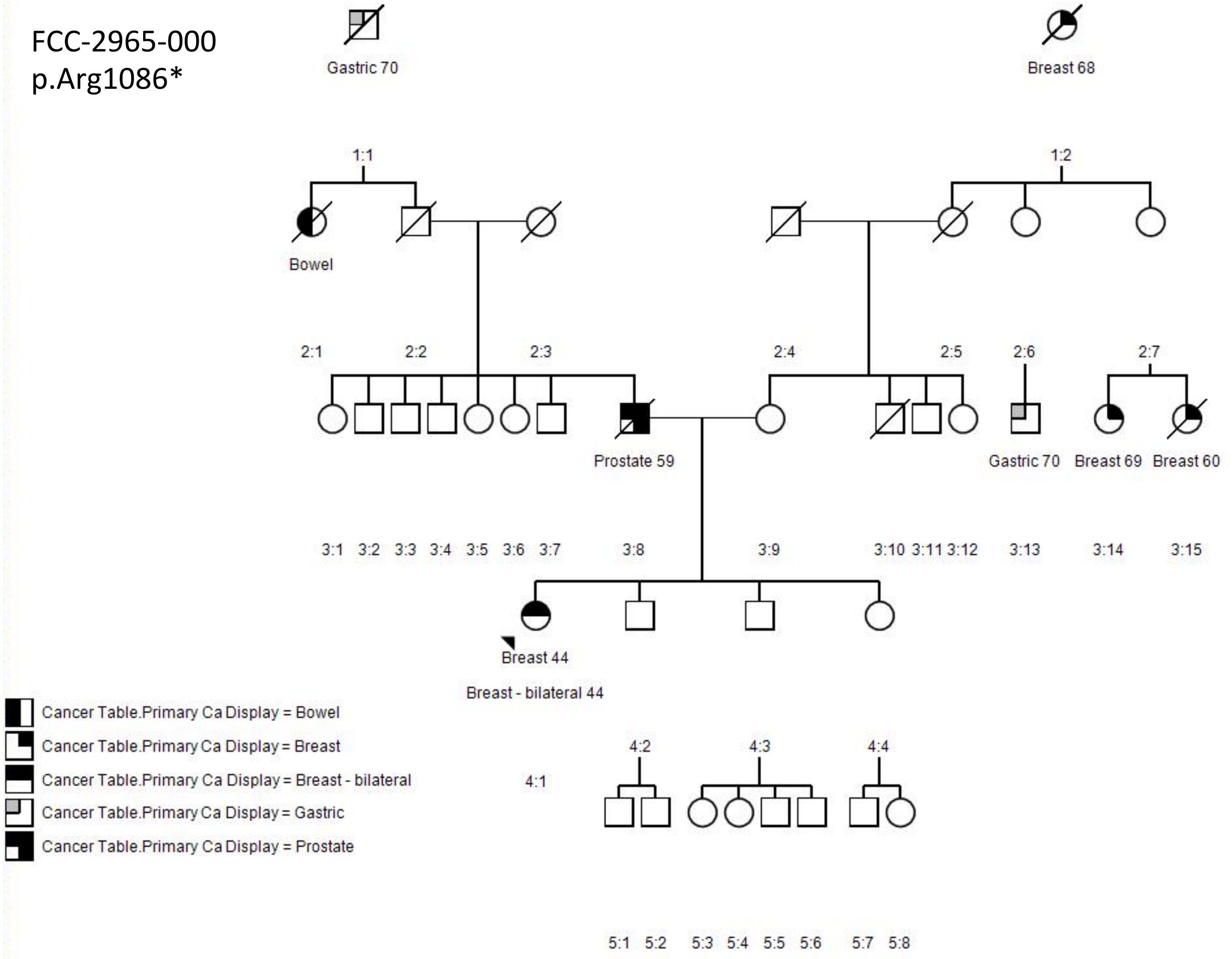

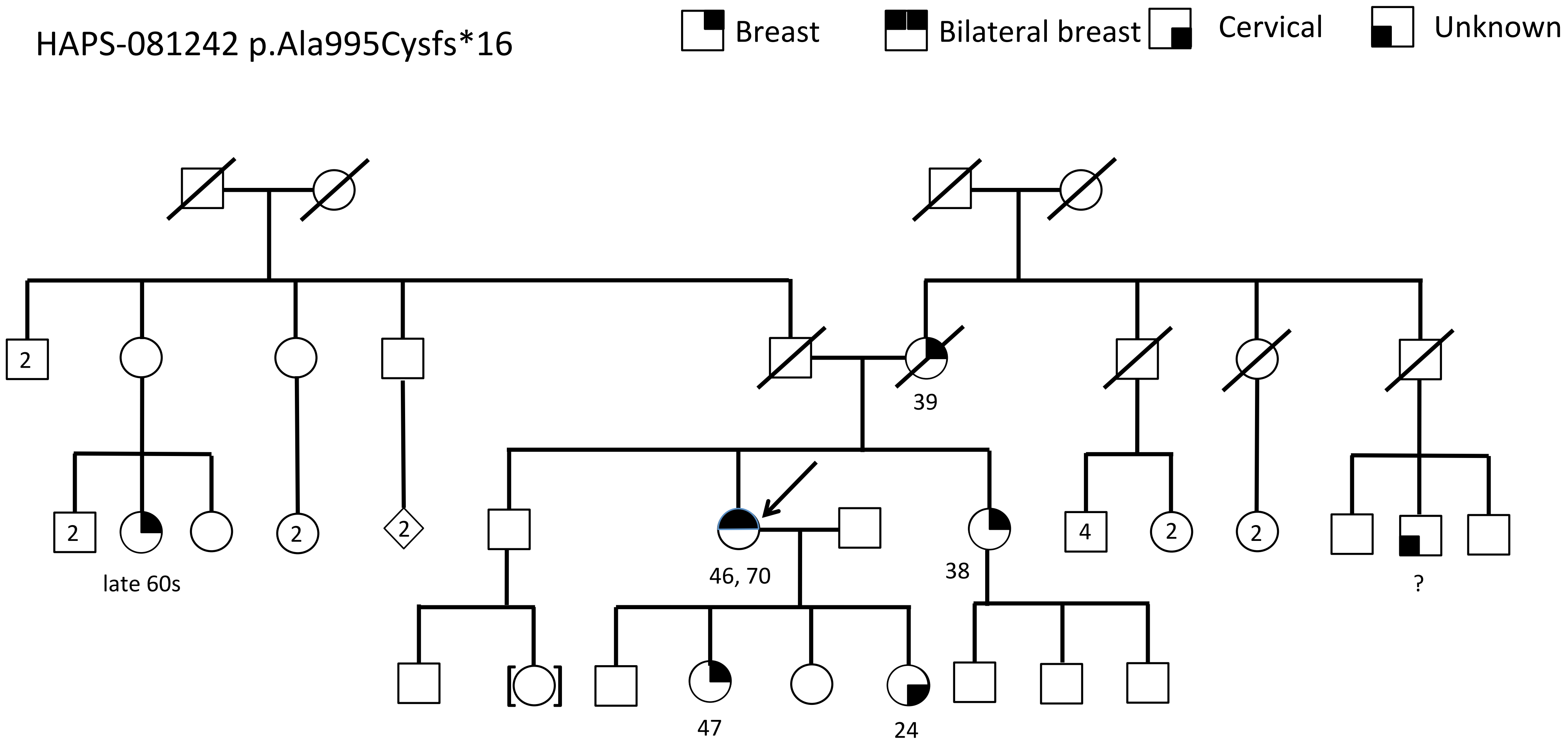

HAPS-081242 p.Ala995Cysfs*16
Breast
Bilateral breast
Cervical
Unknown
2
39
late 60s
2
2
2
46, 70
38
4
2
2
?
47
24

HAPS-060382 p.Gln66*

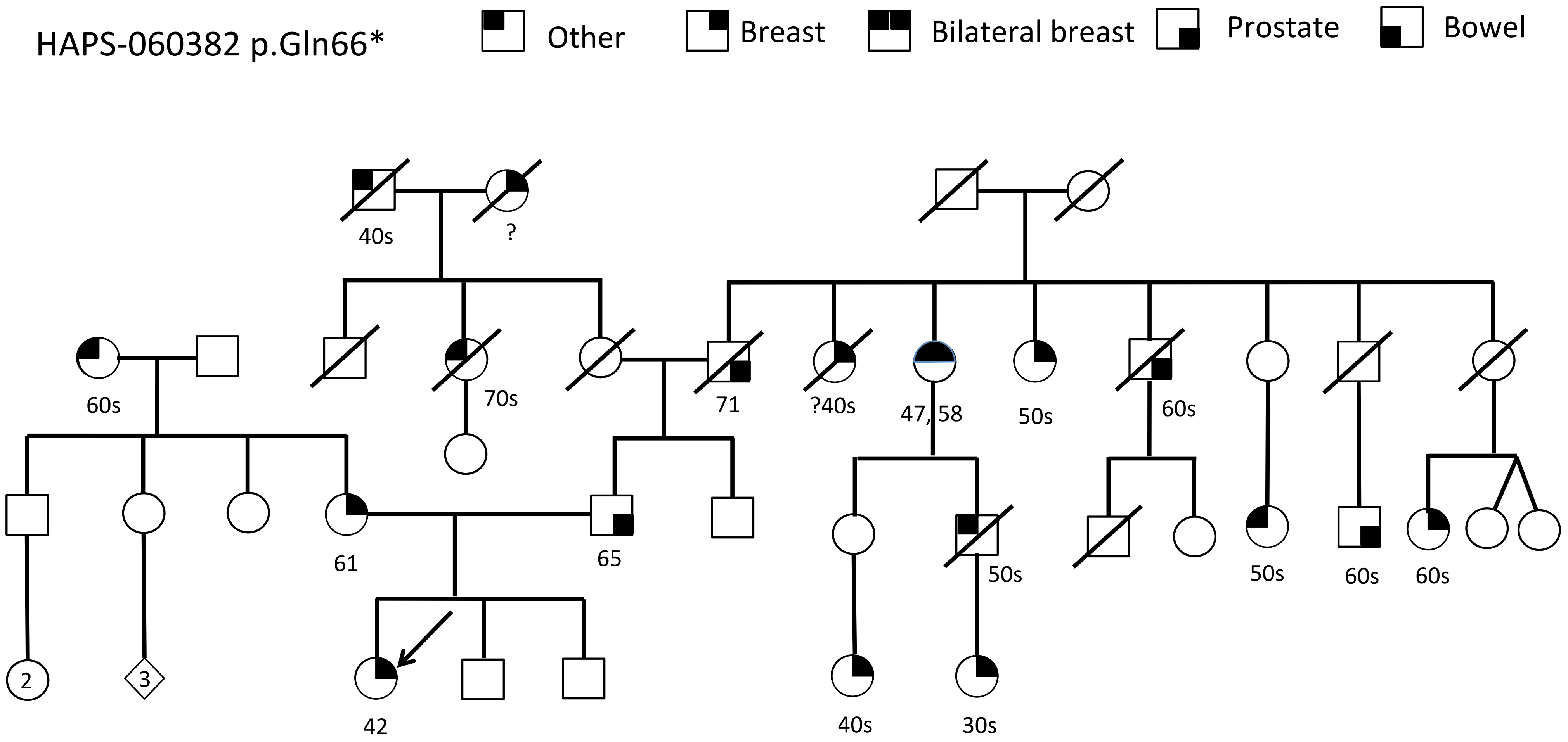

HAPS-110583: p.Tyr1183*

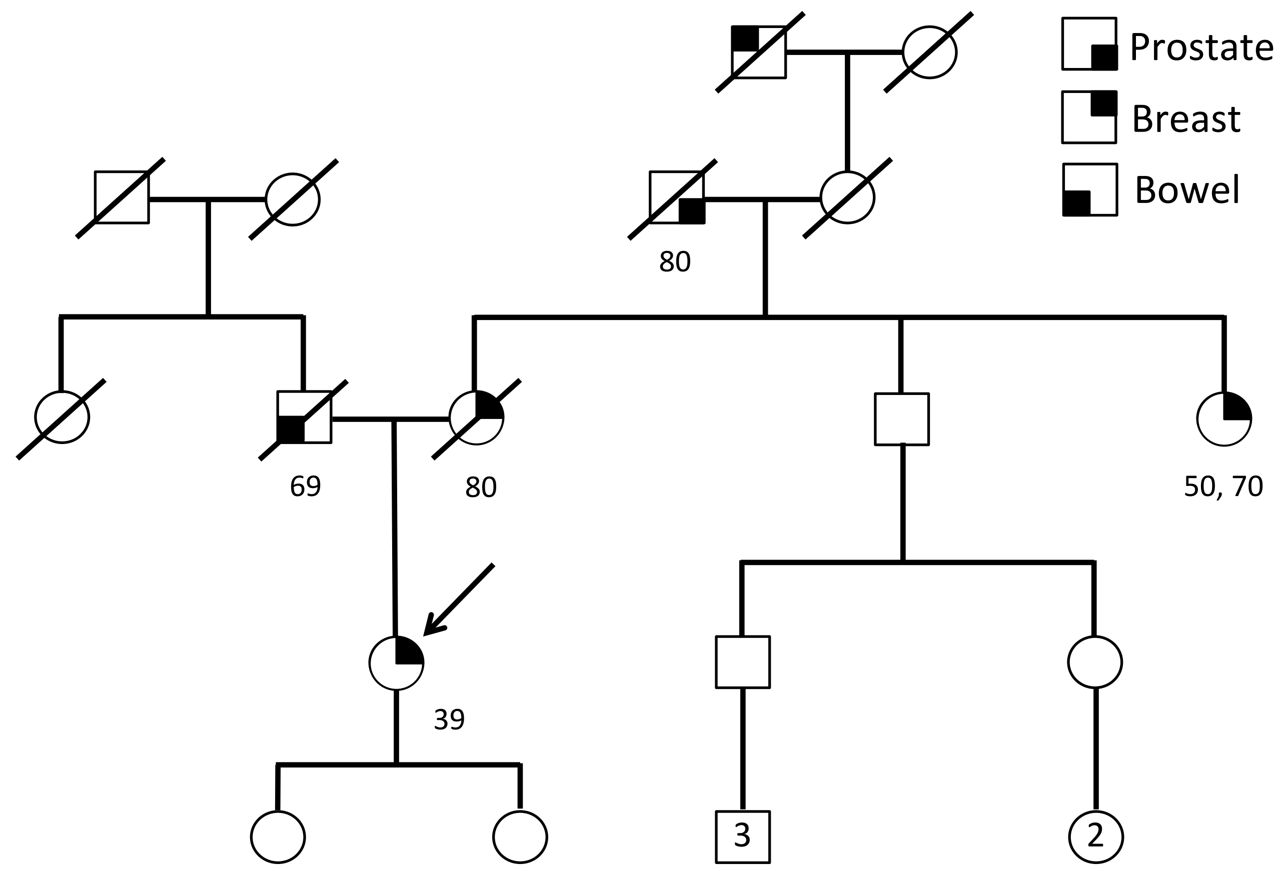

HAPS-090978: *p.Thr193Asnfs*2*

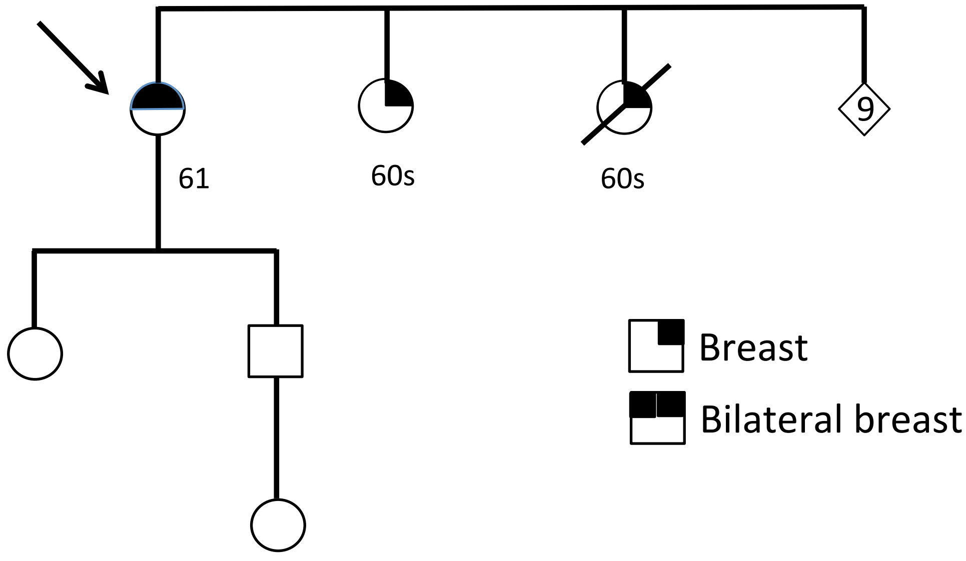

HAPS-100240: p. His1170Phefs*19

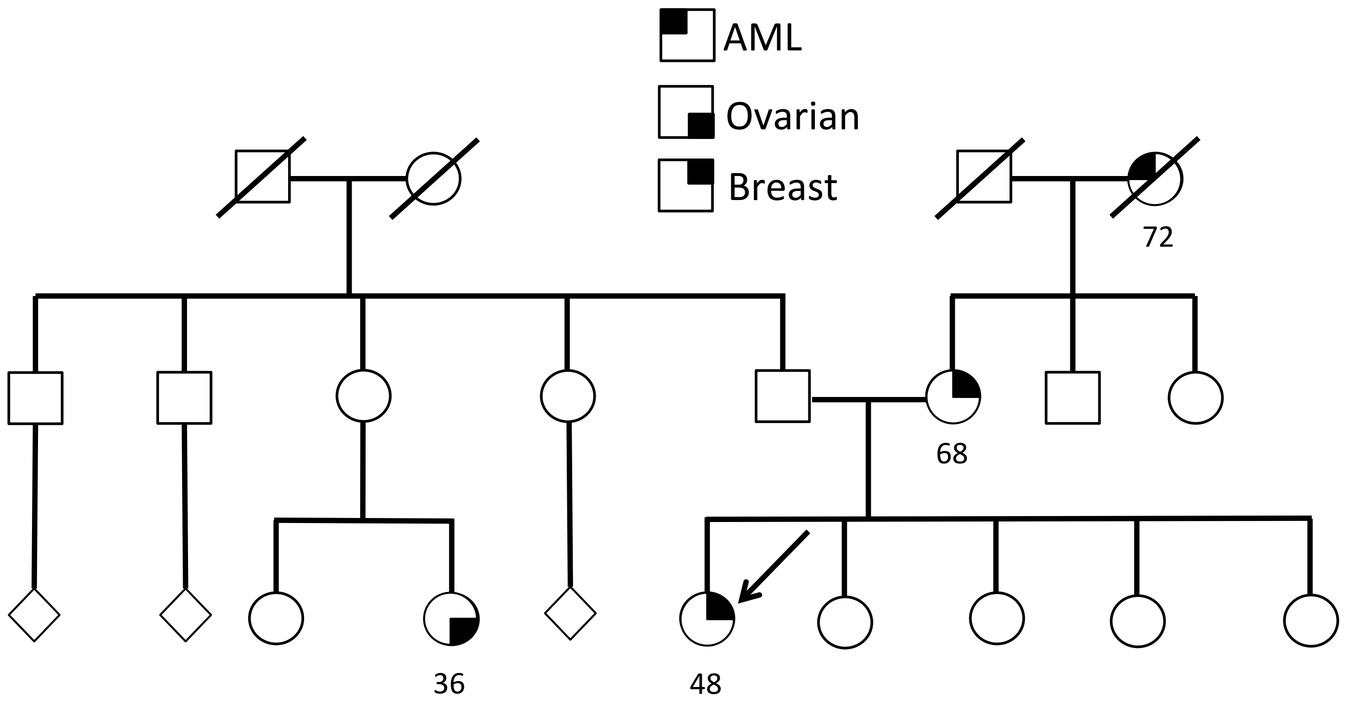

HAPS-110283: p.Trp1038*

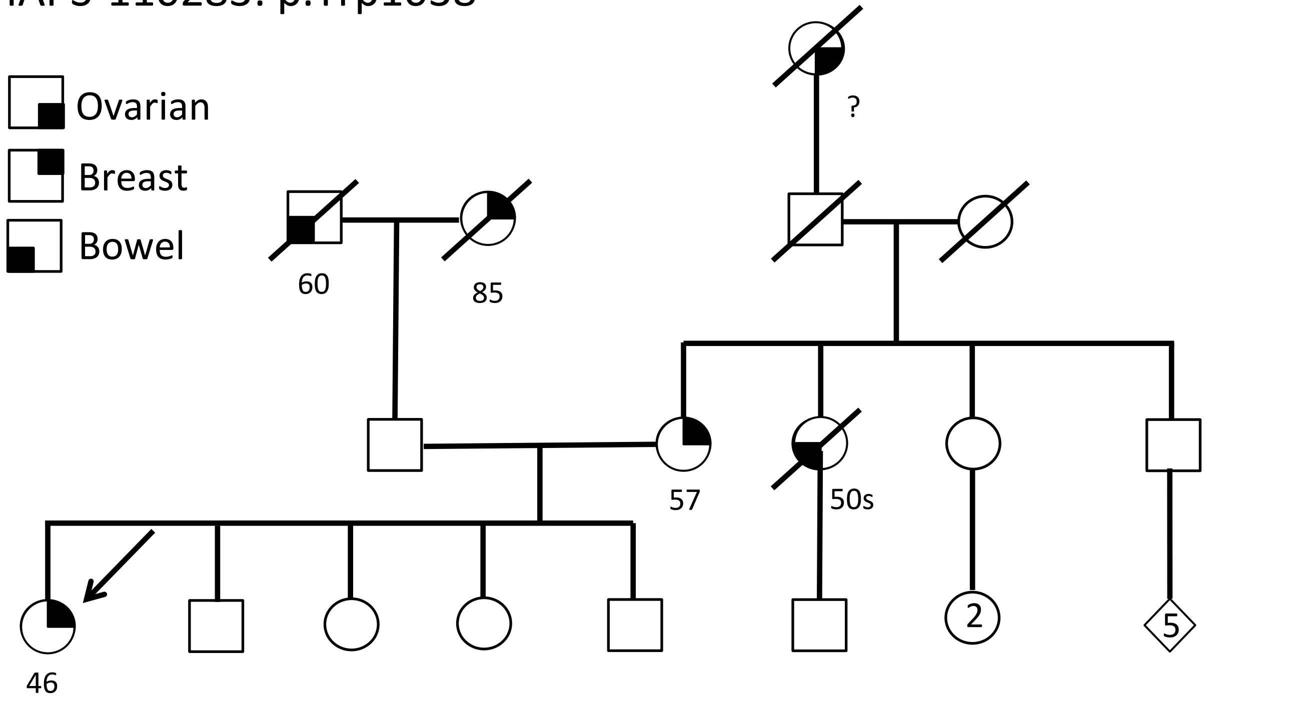

HAPS-121549: p.Trp1038*

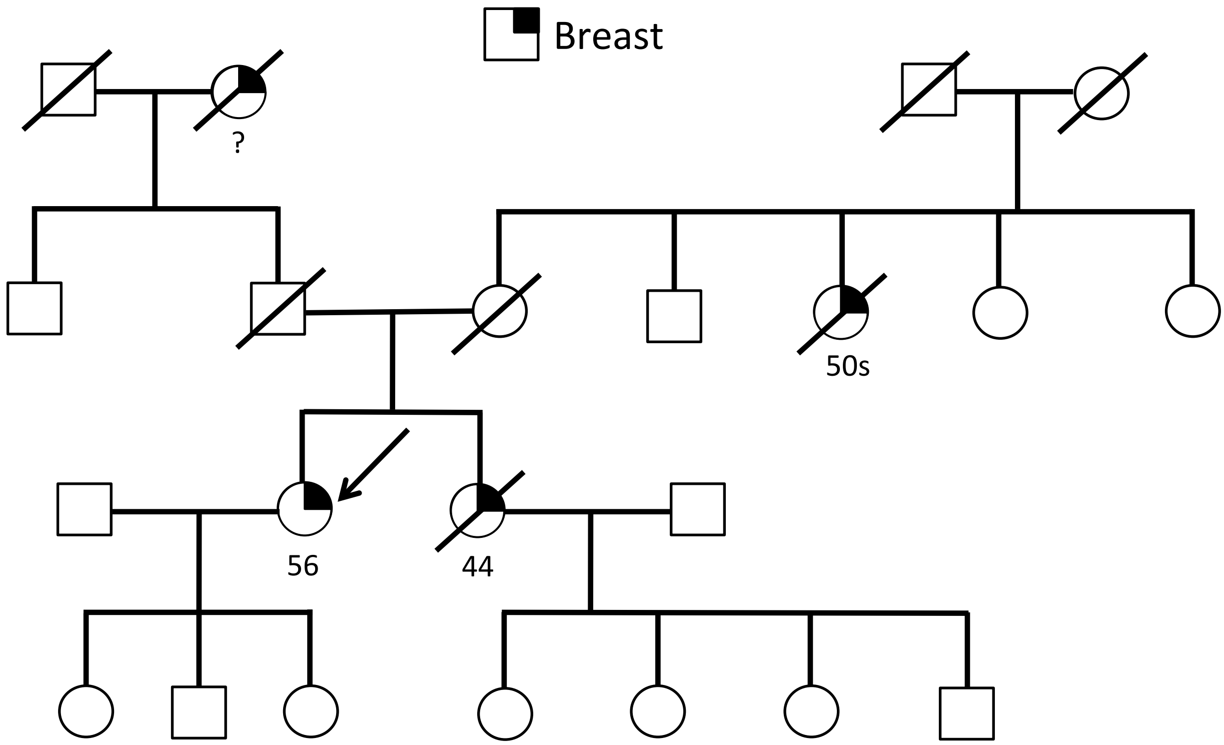

HAPS-114269: *p.Pro656Glnfs*11*

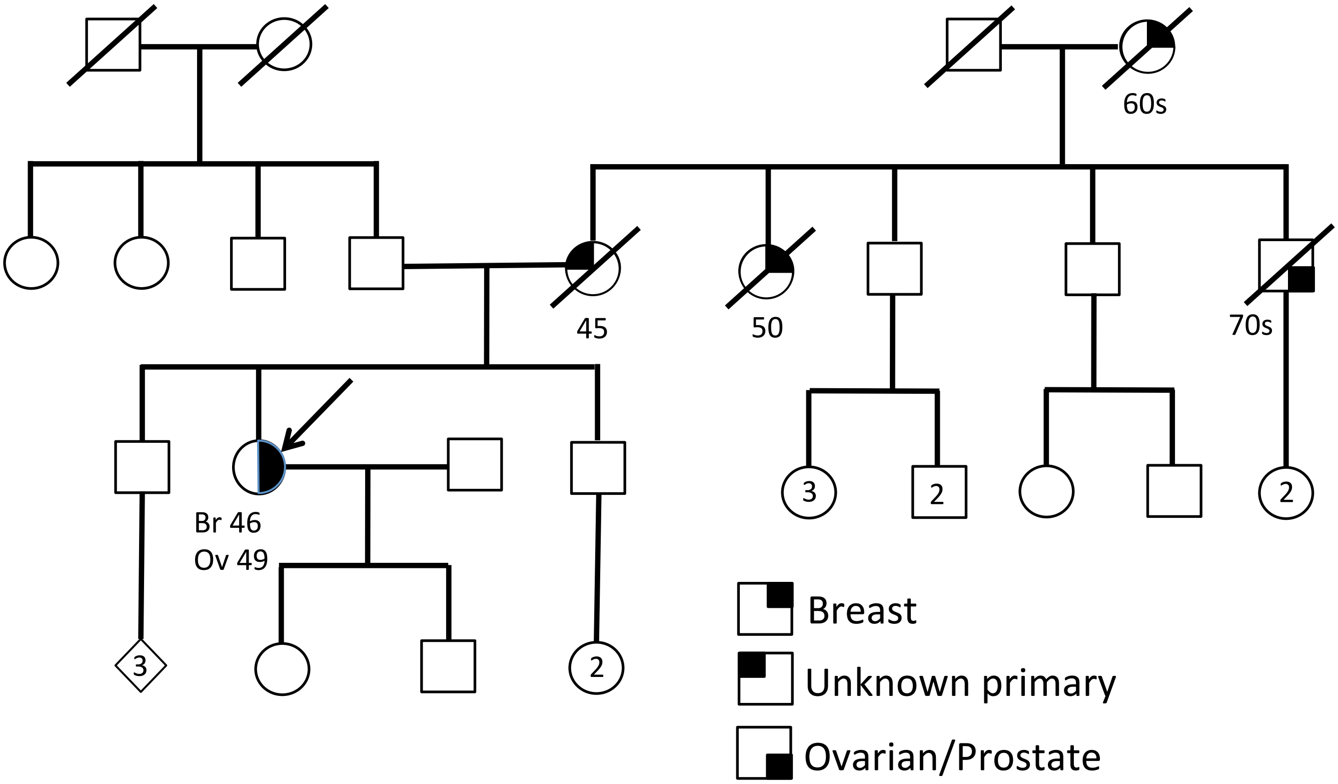

HAPS-120953 p.Gln797Hisfs*54

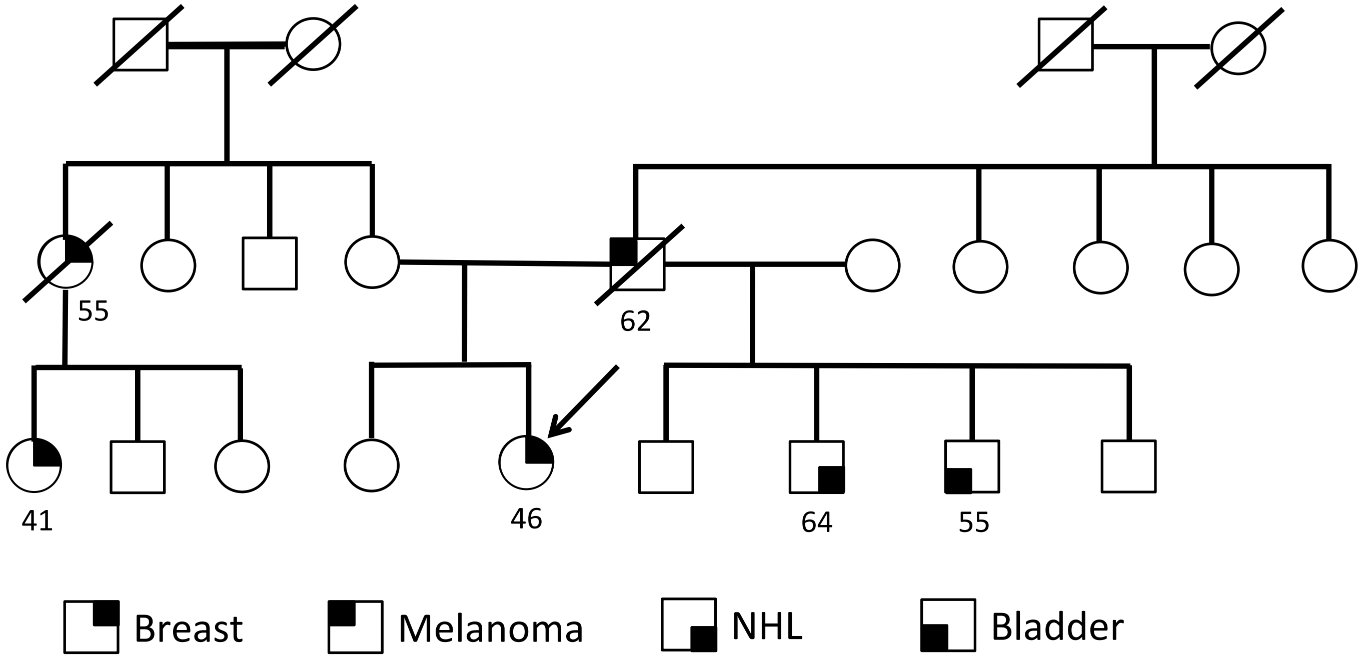

HAPS-120272 p.Ser254Ilefs*3

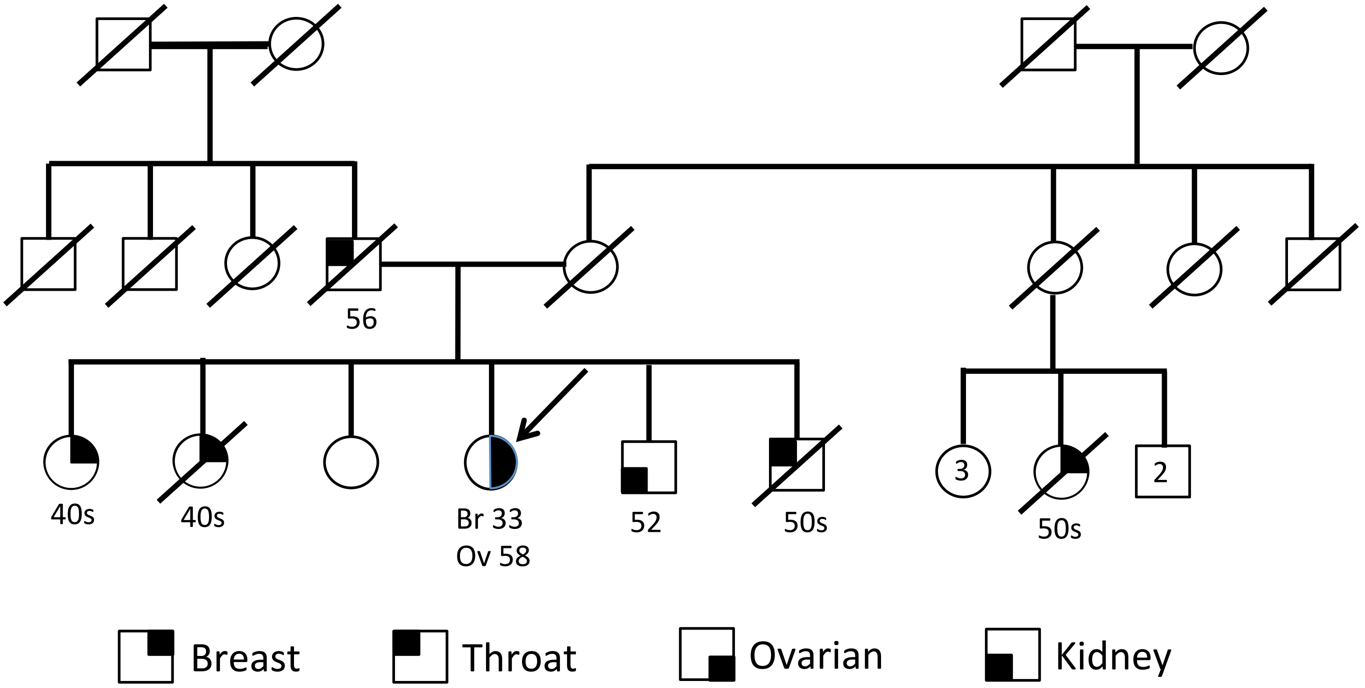

HAPS-090809 p.Gly232Argfs*3

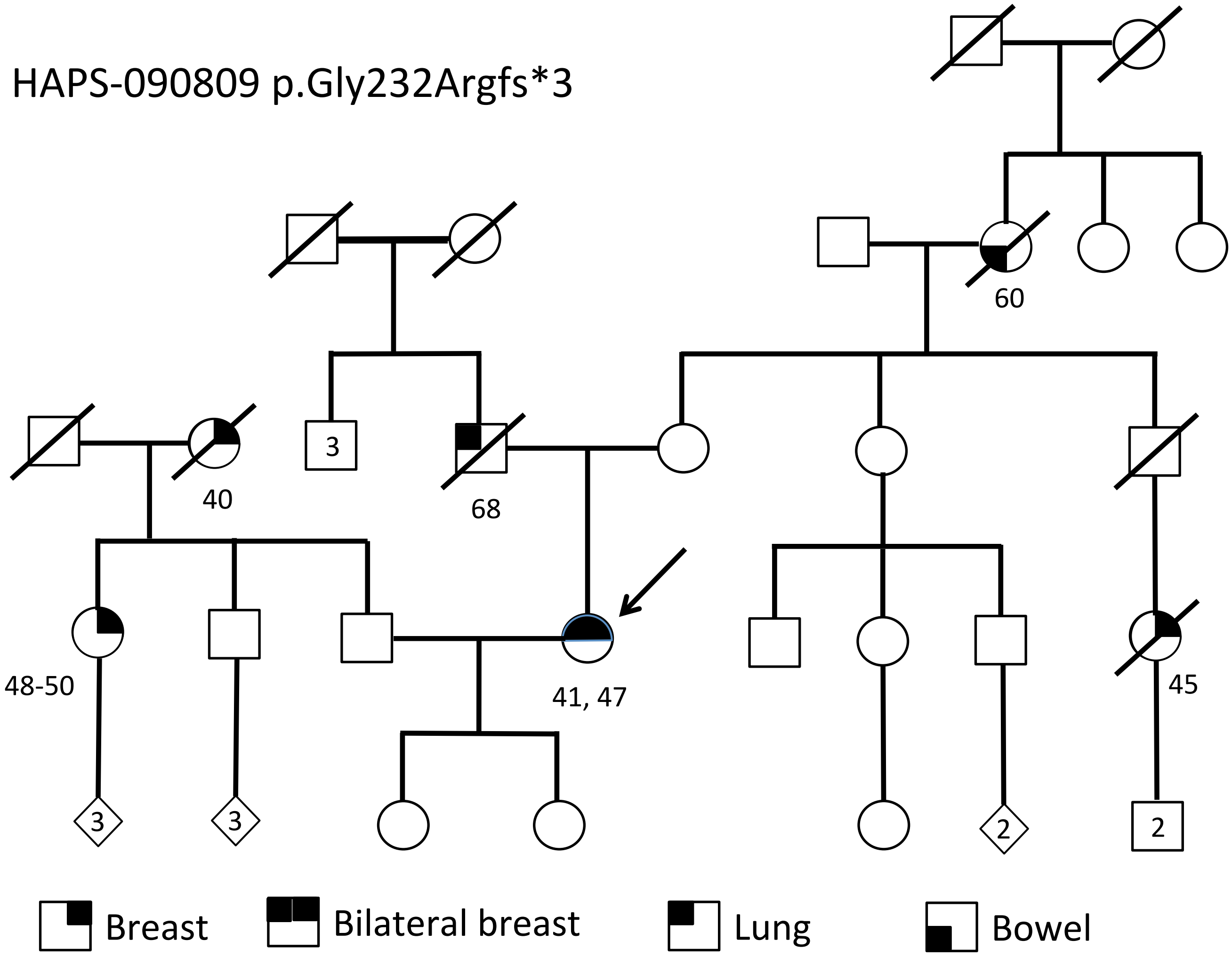

Supplement: Additional file 2: — PALB2 truncating variant carrier family pedigrees (Figure). (PDF 1036 kb) [file 13058_2015_627_MOESM2_ESM.pdf]
